# Assessing Repeated Urinary Proline Betaine Measures as a Biomarker of Usual Citrus Intake during Pregnancy: Sources of Within-Person Variation and Correlation with Reported Intake

**DOI:** 10.3390/metabo13080904

**Published:** 2023-08-02

**Authors:** Caitlin D. French, Charles D. Arnold, Ameer Y. Taha, Reina Engle-Stone, Rebecca J. Schmidt, Irva Hertz-Picciotto, Carolyn M. Slupsky

**Affiliations:** 1 Department of Nutrition, University of California, Davis, CA 95616, USA; cdfrench@berkeley.edu (C.D.F.); cdarnold@ucdavis.edu (C.D.A.); renglestone@ucdavis.edu (R.E.-S.); 2 Department of Food Science and Technology, University of California, Davis, CA 95616, USA; ataha@ucdavis.edu; 3 Department of Public Health Sciences, University of California, Davis, CA 95616, USA; rjschmidt@ucdavis.edu (R.J.S.); iher@ucdavis.edu (I.H.-P.)

**Keywords:** dietary biomarker, citrus, proline betaine, pregnancy, measurement error

## Abstract

Proline betaine (Pro-B) has been identified as a biomarker of dietary citrus intake, yet gaps remain in its validation as a quantitative predictor of intake during various physiological states. This study quantified sources of within-individual variation (WIV) in urinary Pro-B concentration during pregnancy and assessed its correlation with the reported usual intake of citrus fruit and juice. Pro-B concentrations were determined by ^1^H-NMR spectroscopy in spot and 24-h urine specimens (*n* = 255) collected throughout pregnancy from women participating in the MARBLES cohort study. Adjusted linear or log mixed effects models quantified WIV and tested potential temporal predictors of continuous or elevated Pro-B concentration. Pearson or Spearman correlations assessed the relationship between averaged repeated biomarker measures and usual citrus intake reported by food frequency questionnaires. The proportion of variance in urinary Pro-B attributable to WIV ranged from 0.69 to 0.74 in unadjusted and adjusted models. Citrus season was a significant predictor of Pro-B in most analyses (e.g., adjusted *β* [95% CI]: 0.52 [0.16, 0.88] for non-normalized Pro-B), while gestational age predicted only non-normalized Pro-B (adjusted *β* [95% CI]: −0.093 [−0.18, −0.0038]). Moderate correlations (*r_s_* of 0.40 to 0.42) were found between reported usual citrus intake and averaged repeated biomarker measurements, which were stronger compared to using a single measurement. Given the high degree of WIV observed in urinary Pro-B, multiple samples per participant are likely needed to assess associations between citrus consumption and health outcomes.

## 1. Introduction

Commonly used dietary assessment methods relying on self-report, including the 24-h dietary recall (24HDR) and the food frequency questionnaire (FFQ), are subject to substantial recall bias [1,2,3]. This presents analytical challenges for population monitoring and nutritional epidemiological studies by introducing bias into prevalence estimates and regression coefficients and by reducing statistical power to detect diet-health relationships [4,5]. Given these challenges, there is increasing interest in the development and validation of biomarkers to provide unbiased measures of intake of nutrients, foods, food groups, and dietary patterns [6,7,8,9]. Existing dietary biomarkers range from recovery/predictive biomarkers, from which absolute intake during a given time period may be determined based on a known quantitative relationship between the biomarker and intake level [1,5,10], to concentration biomarkers, which although less quantitatively related to absolute intake, are more common and still have proven useful in reducing bias and increasing power to detect diet-health associations [11].

Although dietary biomarkers are not subject to systematic error from recall bias, the use of short-term assessment methods, whether recall- or biomarker-based, to represent long-term usual intake introduces measurement error due to the presence of random, day-to-day variation in dietary intakes within individuals. In addition, many foods and some nutrients (e.g., pre-formed retinol) may be episodically consumed; that is, they are not consumed every day (or nearly every day) by most individuals in a study population. For dietary components falling into this category, the aforementioned challenge of within-individual variation (WIV) is exacerbated by the presence of excess zero values in dietary intake data, which further complicates statistical analyses [12]. In areas of high agricultural productivity and/or where local patterns of consumption of produce are influenced by seasonal variations, episodic consumption and/or WIV in dietary intakes can partially be explained by season [13,14,15,16]. In the case of pregnancy, biological [17,18] or dietary changes [19,20,21] related to gestation progression could introduce an additional source of WIV into biomarker measurements.

Measurement error models developed to account for random WIV in the estimation of a population’s usual dietary intake distributions [22,23,24] and diet-health associations [25,26] have traditionally been applied to repeated 24HDR data but may be useful for biomarker data as well. Urine collections are a minimally invasive method with a relatively low participant burden (especially spot urine collections) that may be beneficial for measuring biomarkers of multiple dietary components from a single sample. Since many measured excretion products are likely to represent short-term intake, similar to 24HDR, repeated sample collections may allow for modeling of usual intake, which is of most interest in nutrition studies. The use of such modeling strategies relies on knowledge of the measurement error structure of the dietary intake data—namely, the within- and between-individual variance components. For episodically consumed dietary components, the probability of daily consumption can be modeled and used in conjunction with the amount consumed on consumption days to model average daily intake [22].

Citrus fruits and juices represent one such dietary component that may be of interest to measure for population monitoring and/or nutritional epidemiological studies due to their high content of essential nutrients such as vitamin C, folate, and fiber, as well as other bioactive phytochemicals that may confer health benefits in humans (reviewed in [27] and [28]). Some epidemiological evidence supports a negative association between consumption of citrus fruit and/or the flavonoids present in them and inflammatory markers in women [29] and ischemic stroke in men [30], and randomized clinical trials have suggested a beneficial effect of orange juice consumption on endothelial function [31,32,33]. Beyond the interest in measuring citrus intake specifically, biomarkers of citrus fruit consumption may serve as an important component of suites of biomarkers that aim to capture general dietary patterns or consumption of specific food groups (e.g., fruits and vegetables) [34,35,36].

Proline betaine (Pro-B), also known as stachydrine, is a plant osmo-protective compound found in high concentrations in citrus fruits and juices that have emerged as a promising biomarker of citrus intake, given its high abundance in, and relatively high specificity to, these foods [37,38,39,40]. A number of exploratory studies have identified Pro-B as a known or potential biomarker of consumption of citrus fruit/juice [41,42,43,44,45,46,47,48], fruit [8,39,49,50], and/or certain healthy dietary patterns [8,49,51,52,53,54]. Dietary intervention studies have provided further validation of Pro-B as a direct biomarker of acute or short-term citrus intake [37,39,55,56]. However, while some researchers have proposed its use as a marker of habitual or long-term citrus intake [46,56,57], excretion profiles of Pro-B after acute intake of orange juice demonstrate that, at least for this dietary source, most Pro-B is excreted within the first 24 or fewer hours [37,39,56], with urinary concentrations peaking between 2 and 6 h and small elevations remaining up to 72 or 96 h after consumption [56]. These data suggest that a single urinary Pro-B measurement should be considered a short- to medium-term biomarker. Thus, it is of interest to understand the degree of WIV that may be expected across repeated urinary Pro-B measurements, which would help clarify the need for multiple measurements per individual to account for this variation when assessing usual intake.

While exploratory metabolomics and acute feeding studies establish urinary Pro-B as an important dietary biomarker, scarce data exist on the quantitative relationship between this largely acute biomarker and reported usual intake in observational studies. Instead, observational studies assessing this quantitative relationship to date have either focused on a relatively short dietary recall period (1–4 days) [39,55,58] or assessed only the relative abundance of Pro-B in biospecimens [45,46,48,57]. One multi-cohort study in pregnant women found correlations between Pro-B levels in serum samples and reported usual consumption of citrus fruit, citrus juice, or combined citrus fruit and juice (correlation coefficients ranging from *r* = 0.29 to *r* = 0.42) [42]. However, to our knowledge, no studies to date have assessed Pro-B measured in urine samples as a dietary biomarker during pregnancy, nor have studies evaluated random day-to-day and other sources of WIV in Pro-B concentrations in pregnant or non-pregnant populations.

Understanding the nature of WIV in this biomarker is essential for accurately modeling long-term average, or usual, levels of citrus intake or Pro-B biological concentrations. The aims of the present study were therefore to (1) quantify within-individual, between-individual, gestational, and seasonal components of variance in urinary Pro-B concentrations during pregnancy, (2) determine the number of specimens required to estimate usual urinary Pro-B and rank individuals on usual Pro-B levels, and (3) determine the correlation between repeated measures of urinary Pro-B and reported usual consumption of citrus foods.

## 2. Materials and Methods

### 2.1. Study Population and Selection of Urine Specimens

This is a secondary analysis of urine samples collected from a nested case-control sample within the Markers of Autism in Babies: Learning Early Signs (MARBLES) cohort, which was conducted in the greater Sacramento area in Northern California, a region where citrus is grown locally. The MARBLES study follows women who had a child with an autism spectrum disorder (ASD) diagnosis prior to enrollment in the study, through their next pregnancy and the first 3 years of the child’s life to identify prenatal and early life environmental and genetic risk factors for ASD. The study design and protocols of the MARBLES study have previously been described in detail [59]. Briefly, extensive questionnaires covering environmental, dietary, and other exposures, as well as biological specimens, were collected from participating mothers and their children. Depending on the time of enrollment, up to 4 urine samples per trimester, including 3 first-morning spot urine samples and one 24-h urine sample, were collected by participants in their homes in sterile containers. Participants were instructed to collect specimens at 1-week intervals, to store spot urine samples in their home freezer until the next study visit, and to collect a 24-h specimen consisting of all urine voids during a 24-h period before the study visit, after which all specimens were transferred to the University of California at Davis and stored at −80 °C until aliquoting/analysis.

For the nested case-control study (*n* = 107 women), spot or 24-h urine specimens collected during pregnancy were retrospectively selected from all participants whose child(ren) born during the study received a diagnosis of ASD (*n* = 32 children) as determined by a data-driven algorithmic method derived from Autism Diagnostic Observation Schedule (ADOS) and Mullen scores [60], or had no developmental concern (NDC, *n* = 79 children) at around 36 months of age (mean, 36.8; range, 34–42). Women whose children were determined to have another developmental concern (e.g., ADHD concerns, speech or language problems, etc.) were excluded from this sub-study. For women meeting the above criteria with urine samples available, urine samples at 4 general time points across pregnancy were selected for NMR analysis using the following protocol. First, pregnancy was divided into 4 temporal quarters, and urine specimens meeting the above criteria were assigned to these quarters. For specimens in each quarter, the average gestational age was calculated and the spot urine sample closest to this average was selected for each participant, for a total of 4 maximum specimens per pregnancy. If no spot urine was available for a given quarter, the closest 24-h urine collection was selected. Spot urine samples were prioritized given the larger number of available samples compared to 24-h urine. In the case that multiple specimens were available for pregnancy but only in one quarter, a second specimen within that quarter that was farthest away (temporally) from the first selected specimen was selected. Due to some mothers being enrolled in the study across more than 1 pregnancy, more than 4 specimens were available for some women, which were all included in the main analysis.

### 2.2. Preparation and NMR Analysis of Proline Betaine Standard

Stachydrine (proline betaine) was purchased from Carbosynth LLC (San Diego, CA, USA). A standard was prepared by combining 11.7 mg of Pro-B with 30 mL of 10 mM phosphate buffer for a final Pro-B concentration of 2.7 mmol/L. ^1^H-NMR spectroscopy was carried out to determine the Pro-B signature for quantifying Pro-B in urine samples. Pro-B standard (207 µL) was combined with 23 µL of DSS-D6 [3-(trimethylsilyl)-1-propanesulfonic acid-d6], an internal standard for quantification of metabolites (Chenomx Inc, Edmonton, AB, Canada), transferred to 3 mm Bruker (Bruker Corporation, Billerica, MA, USA) NMR tubes (*n* = 2 replicates plus 1 phosphate buffer blank sample), and stored briefly at 4 °C. The pH of the samples before data acquisition ranged from 6.83 to 6.85. NMR spectral data were acquired as described previously [61] on a Bruker Avance 600-MHz NMR spectrometer equipped with a SampleJet autosampler (Bruker Corporation, Billerica, MA, USA) using a NOESY-presaturation pulse sequence (noesypr) at 25 °C.

NMR spectra were manually phase- and baseline-corrected using Chenomx NMR Suite v.8.5 Processor (Chenomx Inc., Edmonton, AB, Canada). The Pro-B signature was characterized and saved using Chenomx NMR Suite v.8.5 Spin Simulator, Compound Builder, and Library Manager (Chenomx Inc., Edmonton, AB, Canada).

### 2.3. Quantification of Proline Betaine in Urine Specimens Using ^1^H-NMR Spectroscopy

Urine specimens collected from MARBLES participants between 2007 and 2014 were stored at −80 °C prior to NMR analysis. Before the current experiment, specimens were subjected to 0–3 (median: 1) freeze-thaw cycles for creating aliquots and/or other purposes. Urine specimens were defrosted on ice, centrifuged at 10 K RCF and 4 °C for 10 min, and then tested for the presence of protein using protein urinalysis strips (Uristix^®^, Siemens, Pharmaforte SingaPore Pte, Ltd., Erlangen, Germany). The supernatant (207 µL) was combined with 23 µL of DSS-d6, as described above. Samples were stored at 4 °C for <24-h before adjusting the pH of each sample to 6.85 ± 0.7 by adding small amounts of HCl and/or NaOH. After transferring samples to 3 mm Bruker NMR tubes, NMR spectral data were collected as described above.

After manually correcting the spectral phase and baseline using Chenomx NMR Suite v.8.3 Processor (Chenomx Inc., Edmonton, AB, Canada), small water-soluble compounds and metabolites were identified and quantified using Chenomx NMR Suite v.8.3 Profiler (Chenomx Inc., Edmonton, AB, Canada). Measured concentrations were adjusted for the dilution attributed to the addition of DSS-d6 and, in the case of two specimens with insufficient sample volume, the addition of Milli-Q Ultrapure H_2_O. In cases where it was not possible to visualize two or more peaks associated with the Pro-B signature (due to low concentration or overlapping signals), the sample-specific lower limit of detection (LOD) was determined by fitting the Pro-B profile in Chenomx to the NMR spectrum at 3.1 and 3.3 ppm (the location of the two most prominent peaks associated with Pro-B) to obtain the highest possible concentration for Pro-B within the signal noise in that spectrum. Values determined to be below the LOD were assigned as 0.5*LOD for use in subsequent steps.

### 2.4. Urinary Proline Betaine Variables

All analyses were conducted on non-normalized Pro-B as well as Pro-B normalized to creatinine concentration (µmol/mmol creatinine) for comparison. Given the sensitivity limitations of quantification using NMR leading to a substantial number of samples with undetectable Pro-B, and to consider a potentially lower threshold relevant to recent citrus consumption, the urinary Pro-B data were analyzed in two ways. First, samples with non-detectable Pro-B were assigned values of 0.5*LOD, and Pro-B concentration was analyzed as a continuous variable (Approach 1). As a second approach, Pro-B concentrations below a relevant threshold (100 µM or 30 µmol/mmol creatinine) were assumed to be indicative of not having consumed citrus in the last day—whether due to being completely absent or being present in low amounts from other dietary components or from citrus intake in the more distant past—and assigned as zeros (Approach 2). The 100 µM threshold was chosen by selecting the upper end of previously reported urinary Pro-B concentrations (7.7 ± 6.4 mg/L, which converts to 53.8 ± 44.7 µM) among 26 healthy volunteers after 2 days of abstinence from Pro-B-containing foods [37]. Another threshold previously defined for creatinine-normalized data, 38 µg/mg creatinine [56], was also investigated. After converting from the units used in that report, this threshold was defined as 30 µmol/mmol creatinine.

### 2.5. Assessment of Acute Citrus Consumption

The 24HDR was available for a subsample of participants with corresponding urine samples (*n* = 23). Participants reported all foods and beverages consumed in the 24-h prior to spot urine sample collection (*n* = 20) or coinciding with 24-h urine collections (*n* = 3). Since full 24HDR was only collected at the beginning of the study before a protocol change, the subsample of pregnancies with available 24HDR all occurred between 2007 and 2008. Reported consumption of any citrus foods or juices on the previous day was analyzed as a dichotomous variable and used to assess agreement with the selected thresholds for urinary Pro-B of 100 µM or 30 µmol/mmol creatinine as indicative of recent citrus consumption. When cases arose in which Pro-B concentration exceeded the threshold but no dietary citrus consumption was reported, items that may contain citrus (e.g., “fruit juice”) were investigated, along with other potential dietary sources of Pro-B. The latter included less commonly consumed food sources or those with a lower Pro-B content than citrus, including gorgonzola cheese, kiwi, pineapple, grapes, seafood, shrimp, mussels, Chinese artichoke (*Stachys affinis*), rye or whole-wheat products, alfalfa sprouts, capers, and chestnuts [37,39,62,63,64,65,66].

### 2.6. Assessment of Usual Citrus Consumption

A semi-quantitative food frequency questionnaire (FFQ) (Block 2005, NutritionQuest, Berkeley, CA, USA) was conducted 1–2 times per pregnancy with each participant. The original recall period of the FFQ (one year) was modified by way of written and/or verbal instructions to participants to capture diet from the first or second half of pregnancy. Three items on the FFQ pertained to the frequency and quantity of citrus consumption including (1) oranges or tangerines, (2) grapefruit, and (3) 100% orange or grapefruit juice. The frequency of consumption of each item was converted into frequency per day units. The daily probability (frequency) of consuming any of the three items was determined by the formula

(1) $$\begin{array}{ll} Pr(O\;or\;G\;or\;OJ) & \\ & =Pr(O)+Pr(G)+Pr(OJ)-Pr(O\;and\;G)\\ & -Pr(O\;and\;OJ)-Pr(G\;and\;OJ)+Pr(O\;and\;G\;and\;OJ) \end{array}$$

where O is orange or tangerine consumption, G is grapefruit consumption, and OJ is orange or grapefruit juice consumption. The average total daily citrus servings consumed was estimated by multiplying each citrus item’s daily frequency by the reported amount usually consumed and summing the three items. Servings were defined as approximate cup equivalents (described in more detail in the Supplemental Materials, Table S1). Reported citrus intakes from multiple FFQ time points were averaged to obtain a measure of usual citrus consumption throughout the entire period of urine sample collections.

### 2.7. Predictors and Covariates

The citrus season was defined as urine specimen collection occurring between the months of December and May, the approximate time during which citrus fruits are in season in the study area. Gestational age at the time of specimen collection was determined based on the last menstrual period or ultrasound. Demographic and medical information were collected via structured phone interviews, self-administered questionnaires, and/or medical records [59].

Maternal metabolic conditions were categorized as follows: (1) healthy weight with no metabolic conditions, (2) overweight with no metabolic conditions, (3) obesity with no other metabolic conditions, (4) hypertension/preeclampsia without diabetes mellitus, and (5) type 2 diabetes and/or gestational diabetes with or without hypertension/preeclampsia.

### 2.8. Statistical Analyses

Within-individual predictors of Pro-B concentrations (approach 1) and estimation of variance components: Variance components of urinary Pro-B concentrations treated as a continuous variable were calculated using linear mixed-effects regression models. Within- and between-individual variance components were determined as previously described [67], including Pro-B as the response variable and a random intercept for the person-specific effect. For adjusted analyses, time-dependent predictors tested for inclusion included citrus season (binomial) or four seasons (categorical) and gestational age (months); individual-level factors included maternal age, race/ethnicity, height, weight, education level, gestational diabetes, and other metabolic conditions; and technical/biological confounders included urinary creatinine concentration (µM), urine specimen collection method (24-h or spot), and storage time (days). Those found to be significantly associated (*p* < 0.10) with Pro-B in univariate models were considered for inclusion in adjusted analyses. Collinearity of predictors was assessed by testing for significant univariate associations (*p* < 0.05) in mixed effects models. Urinary creatinine was included as a covariate in non-normalized analyses only. From adjusted models, the residual within- and between-individual components of variance were determined. The proportion of variance attributable to the fixed effects predictors (citrus season and gestational age at the time the sample was collected) were obtained by calculating the marginal r-squared of the model using the R statistical package “MuMIn” (version 1.43.17) [68]. Pro-B concentrations (and other variables where necessary) were log-transformed prior to analysis to improve the assumptions of normal residuals and, in the case of adjusted analyses, linear relationships between independent variables and modeled predictions. To determine if the inclusion of multiple pregnancies per participant influenced the estimation of variance components and/or the association with gestational age, analyses were repeated after (1) including only the first pregnancy during the study for each participant (*n* = 247 samples), and (2) randomly selecting a subset with the same number of samples (*n* = 247).

Within-individual predictors of elevated urinary Pro-B and relation with reported daily frequency of citrus consumption: The overall proportion of elevated urinary Pro-B (≥100 µM or ≥30 µmol/mmol creatinine) was described for the whole sample and by the citrus season. Time-dependent and individual-level predictors of elevated Pro-B, along with potential technical/biological confounders, were tested using mixed-effects logistic regression models. Models included a random intercept effect to account for repeated measures, and ten points per axis were used to evaluate the adaptive Gauss-Hermite approximation to the log-likelihood. The same time-dependent, individual, and technical/biological predictors as described above for the continuous Pro-B variable (approach 1) were tested for inclusion in adjusted models using univariate (plus random effects) models. Continuous predictors were scaled as needed to avoid model convergence problems. The assumption of linearity between model predictors and log odds was inspected visually using Loess plots.

Number of samples required to estimate usual urinary proline betaine of individuals: Within- and between-individual variance components in urinary Pro-B were used to determine the number of repeated measures required to estimate long-term average (usual) Pro-B levels using the formula from Beaton et al. [69]:

(2) $$n=\frac{Z_{\alpha }{CV}_{W}}{D_{0}}$$

where *n* is the required number of measurements per individual, *Z*_α_ is the standardized value for the percentage of times the averaged measured values should fall within the specified limit, *CV_w_* is the within-person coefficient of variation, and *D*_0_ is the specified degree of error as a percentage of true long-term average excretion. A range of *D*_0_ values (5–20%) and a *Z*_α_ of 1.96 were used in the calculations. For the creatinine-normalized data, the *CV_w_* was derived from a mixed effects model after adding a constant of 1 and log transforming the resulting data to avoid negative values in the data used for the *CV_w_* calculation.

To determine the number of samples required to rank individuals on usual Pro-B levels with different levels of accuracy, the following formula from [70] was used:

(3) $$n=\left(\frac{r^{2}}{1-r^{2}}\right)\left(\frac{s_{w}^{2}}{s_{b}^{2}}\right)$$

where *n* is the required number of measurements per individual, *r* is the correlation level, and *s^2^_w_*/*s^2^_b_* is the ratio of within- to between-individual variance in Pro-B. Values of *r*^2^ ranging from 0.5 to 0.9 (corresponding to *r* values 0.71–0.95) were used.

Correlation between urinary proline betaine and reported usual citrus consumption: Mixed effects models as described above for continuous Pro-B or the probability of elevated Pro-B were conducted to determine the association between average FFQ-reported usual citrus consumption (daily servings or probability of consumption, respectively) and this biomarker accounting for random day-to-day variation in repeated measures. To address skewness in the data, reported citrus intakes were naturally log-transformed after adding a constant equal to the minimum nonzero intake value multiplied by 0.75 to remove zero values. To determine the level of agreement between repeated biomarker measures and reported usual intake, Pro-B concentrations (approach 1) were averaged within individuals to obtain the best available measure of individual usual Pro-B exposure, and Pearson or Spearman correlations were calculated, as appropriate. To determine the influence of the number of repeated samples per individual on observed correlations, correlation analyses were repeated after subsetting the data to include *n* = 1, *n* = 2, *n* = 3, or *n* = 4 samples per person. Due to varying numbers of available specimens per participant, sample sizes varied for each of these analyses; thus, correlations using 1, 2, 3, or 4 repeated measures (as available) were repeated on 2 subsets, including (1) individuals with ≥3 samples available (*n* = 38), and (2) individuals with ≥4 samples available (*n* = 13).

All analyses were conducted in R Statistical Software version 4.1.0 or 4.3.0 (R Core Team 2021, 2023).

## 3. Results

### 3.1. Participant and Specimen Characteristics

The study participants included in this analysis were pregnant women aged 34.4 ± 5.1 years. The demographic characteristics and medical conditions of the participants are summarized in Table S2 (Supplemental Materials). Characteristics of 255 urine specimens from the 107 women are summarized in Table 1. Most (85%) of the specimens were first-morning spot urine collections, while 15% were 24-h collections. Urine sample storage times before NMR analysis ranged from 1572 to 4198 days. The median number of previous thaws before the present analysis was 1 (*n* = 217), with a maximum of 3 previous thaws (*n* = 1). Most samples (92.5%) were collected during the second or third trimester of pregnancy (see Table 1 and Figure S1 in the Supplemental Materials).

### 3.2. Urinary Proline Betaine Concentrations

Pro-B was not detectable in 22% (*n* = 57) of the NMR spectra, while 2 or more peaks consistent with the Pro-B signal were identified in the remaining samples (Table S3, Supplemental Materials). Urinary Pro-B distributions for non-normalized and creatinine-normalized continuous data (approach 1) are displayed in Table 2. The number of urine specimens per participant ranged from 1 to 5. Among those with 2 samples available, which represented most participants, 56% had elevated Pro-B (>100 µM) in at least one sample (Table S4, Supplemental Materials).

### 3.3. Agreement between 24HDR and Urinary Proline Betaine Relevant Threshold for Recent Citrus Consumption

**Figure 1:**
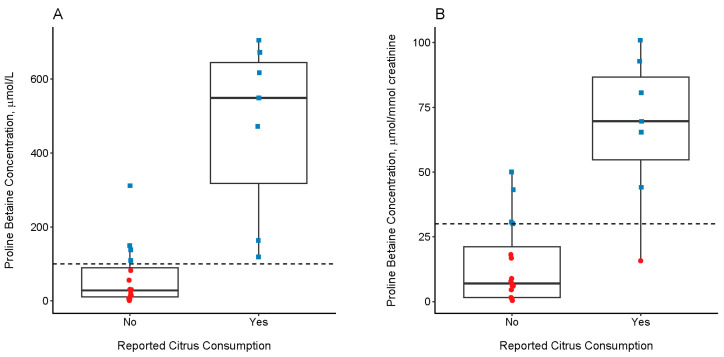
Urinary proline betaine (Pro-B) concentrations in a subset of MARBLES participants, by 24-h, reported citrus consumption. Dashed lines represent the thresholds above which citrus consumption in the previous 24-h is assumed. Red circles represent Pro-B concentrations below the threshold; blue squares represent Pro-B concentrations above the threshold. (**A**) Non-normalized Pro-B concentrations, using a threshold of 100 µM. (**B**) Pro-B concentrations normalized to urinary creatinine, using a threshold of 30 µmol/mmol creatinine.

Twenty-three 24HDR from 14 participants were available for comparison to spot urine specimens collected the morning following the 24-h recall period (*n* = 20) or 24-h urine specimens collected throughout the recall period (*n* = 3). Using non-normalized Pro-B data, an agreement between citrus consumption reported in the 24HDR and urinary concentrations above 100 µM was 82.6%. Specifically, all 7 recalls indicating citrus consumption had corresponding urinary Pro-B concentrations above the 100 µM threshold, while 4 of the 16 24HDR that did not report citrus consumption had Pro-B concentrations above 100 µM (Figure 1A). The relevant threshold defined for creatinine-normalized Pro-B, 30 µmol/mmol creatinine, was in similar agreement with the 24HDR (78.3%), with one sample corresponding to reported citrus consumption falling below the threshold and 4 samples not reporting consumption falling above the threshold (Figure 1B). Upon further investigation, for one sample with clearly elevated Pro-B by either threshold but not reporting citrus explicitly, the participant had reported consuming “fruit juice”. For another such sample falling just above either threshold, it was discovered the participant had consumed guacamole likely containing a small amount of citrus juice. Agreement between the two thresholds’ classification of specimens was 87%.

### 3.4. Temporal Predictors of Urinary Proline Betaine

Continuous variable: Two potential sources of within-person variability in urinary Pro-B concentration were tested as predictors in linear mixed effects models. On average, citrus season (December–May) at the time of urine collection was associated with 68% to 72% higher Pro-B in unadjusted models (Table 3). After adjustment for covariates, this association remained statistically significant and accounted for an estimated 3% of the overall variance in Pro-B concentrations (Table 4). In contrast, gestational age was significantly associated with non-normalized urinary Pro-B but not with Pro-B normalized to urinary creatinine and accounted for less variance based on the r-squared for the model (Table 4). To investigate creatinine as a possible confounder of the relationship between non-normalized Pro-B and gestational age, the association between creatinine concentration and gestational age was examined and found to be not statistically significant (*p* = 0.16, marginal R^2^ = 0.006). Covariates in adjusted models included citrus season, gestational age, creatinine, and, in the case of creatinine-normalized data, metabolic conditions. In the creatinine-normalized analysis, a potential multi-collinearity issue was detected between specimen gestational age and one metabolic condition (obesity without the presence of other metabolic conditions), as these were significantly associated (*p* = 0.046). However, the association between Pro-B and gestational age did not change meaningfully when observations (*n* = 40) from individuals reporting this condition were excluded, nor were results meaningfully different between adjusted models including either gestational age or metabolic conditions or both. Excluding data from the second pregnancy in cases of multiple pregnancies resulted in similar associations between Pro-B and gestational age.

Probability of elevated proline betaine: The influence of citrus season and/or gestational age on the probability of elevated urinary Pro-B, using thresholds likely to indicate recent citrus consumption, was also investigated. The overall proportion of specimens with elevated urinary Pro-B was 0.42 (>100 μM) or 0.35 (>30 µmol/mmol creatinine) in the whole sample and varied between specimens collected during the citrus season (December–May) versus those collected between June and November (Table 5). After accounting for repeated measures using mixed effects logistic regression, the association between elevated Pro-B and the citrus season was not statistically significant at the α = 0.05 level in unadjusted (OR = 1.72, CI: 0.94–3.15, *p* = 0.08) or adjusted models when considering the non-normalized cutoff (Table 5). However, this association was found to be statistically significant (*p* = 0.04) when considering the creatinine-adjusted threshold (Table 5).

Gestational age at the time of sample collection was not a significant predictor of elevated Pro-B, regardless of whether the >100 μM (adjusted *OR* = 0.91, CI: 0.78–1.07, *p* = 0.26) or >30 µmol/mmol creatinine (adjusted *OR* = 0.92, CI: 0.77–1.09, *p* = 0.32) cutoff was considered.

### 3.5. Within- and between-Individual Variance Components of Urinary Proline Betaine

Continuous variable variance components: From the linear mixed effects regression models described above, within- and between-individual components of variance in non-normalized or creatinine-normalized urinary Pro-B concentrations were quantified and are shown in Table 6. The proportion of total variance in Pro-B attributable to WIV ranged from 0.69 to 0.74, depending on the normalization method and whether covariates were accounted for before partitioning the residual variance (Table 6). The inclusion of covariates in the model was slightly more influential on this proportion in non-normalized data than in creatinine-normalized data, but overall, the estimates were relatively stable across methods. Sensitivity analyses indicated that the proportion of variance attributable to WIV was higher among spot urine samples than in the combined sample, in particular for non-normalized Pro-B unadjusted for covariates (WIV:total = 0.86) (Table S5, Supplemental Materials). Excluding data from the second pregnancy of multiple pregnancy cases resulted in slightly lower WIV:total ratios, which were more pronounced for non-normalized data (maximum difference = 0.08, unadjusted WIV:total). However, excluding a random sample of the same size resulted in similar differences in most cases.

Variance components of “nonzero” data for use in 2-part models: Dietary data with many zero intake observations may be analyzed using a 2-part model to jointly model (1) the probability of occurrence, and (2) the amount on occurrence days, the latter of which is adjusted for WIV in usual distribution and regression calibration models. Within- and between-individual components of variance were therefore quantified in the portion of urinary Pro-B concentrations falling above the relevant thresholds used in the probability models described above (>100 µM or >30 µmol/mmol creatinine). As expected, WIV and BIV were both substantially reduced compared to these variance components in the continuous data used in approach 1. For non-normalized data above the threshold, variance ratios (Table 7) were slightly higher than those found for the approach 1 data (Table 6). However, for creatinine-normalized data, variance ratios were much lower, which was explained by larger relative reductions in WIV than in BIV (Table 7). Neither citrus season nor gestational age were statistically significant predictors of urinary Pro-B concentration in this subset of observations with elevated Pro-B (*p* > 0.10, Table S6, Supplemental Materials) and were thus not included in adjusted analyses.

### 3.6. Number of Samples Required to Estimate Usual Urinary Proline Betaine

The within- and between-individual components of variance in urinary Pro-B were used to calculate the number of repeated samples that would be required to (1) estimate true average, long-term (usual) Pro-B levels of individuals, and (2) rank individuals on usual Pro-B levels with varying degrees of accuracy. As shown in Table 8, the number of samples required for either purpose decreases with higher allowed levels of uncertainty (less precision). The number of samples required to estimate true usual creatinine-normalized Pro-B was higher than for non-normalized Pro-B due to the higher *CV_w_* for the former. However, when the goal is to rank individuals on Pro-B levels, the required number of samples is similar regardless of normalization to creatinine, given similar WIV:BIV ratios.

### 3.7. Level of Agreement between Urinary Proline Betaine and Reported Usual Citrus Consumption

The distribution of reported usual citrus intake based on averaged FFQ responses was right skewed, with a median intake of 0.32 (IQR, 0.50) citrus servings/day. The distribution of the reported daily frequency of citrus consumption was also right-skewed, with a median frequency of 0.29 (IQR, 0.35).

**Figure 2:**
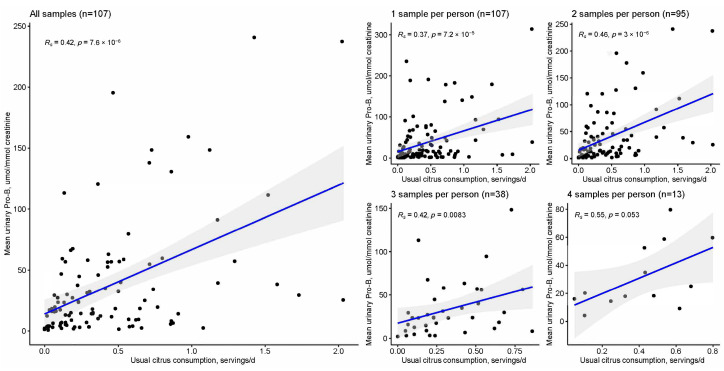
Scatter plots of urinary proline betaine concentration (µmol/mmol creatinine) by reported usual citrus intake (servings/day), comparing single sample data with averaged repeated measures. *R_s_* represents the Spearman’s rank correlation coefficient. Blue lines are derived from linear regression and are included only to visualize trends in the data.

Continuous variable: In mixed-effects models accounting for a repeated spot or 24-h urinary measures, usual citrus consumption reported by FFQ was strongly associated with non-normalized (β = 0.56, 95% CI: 0.38–0.75, *p* < 0.0001) and creatinine-normalized (β = 0.60, 95% CI: 0.42–0.78, *p* < 0.0001) urinary Pro-B on log-transformed scales. When urinary concentrations were averaged among repeated measures within participants, average Pro-B concentration was moderately correlated with reported citrus consumption, with generally stronger correlations found after normalization to creatinine (Table 9). Varying the number of urinary measurements per person influenced the strength of the correlation between reported intake and creatinine-normalized Pro-B levels. Except for the subsample including 3 measures per individual, successively higher correlation coefficients were found with increased numbers of samples per participant despite decreasing available sample sizes with higher numbers of repeats (Figure 2). Log transforming both the FFQ and Pro-B variables improved the linearity and strength of the correlations for the first three subsets, which included 1, 2, or 3 samples per individual, but did not meaningfully alter the correlation when the data was subset to include 4 samples per individual (Figure S2, Supplemental Materials). Given varying numbers of individuals with 2, 3, or 4 repeated measurements available and in order to compare correlations within a consistent study sample, analyses were repeated on (1) the subset of individuals with ≥3 samples available, and (2) the subset of individuals with ≥4 samples (Figures S3 and S4, Supplemental Materials). In the latter analyses, the strongest correlation was observed for *n* = 2 or *n* = 3 averaged samples, respectively.

Probability of elevated proline betaine: The level of agreement between the reported frequency of citrus fruit or juice consumption and the probability of elevated Pro-B was also examined. The overall probability of elevated Pro-B among all samples was comparable to the mean daily frequency of citrus consumption based on FFQ responses (mean = 0.34) when using the creatinine-normalized cutoff (proportion = 0.35) but less so when using non-normalized data (proportion = 0.42), and these proportions were less comparable to the median reported daily frequency of 0.29. In unadjusted and adjusted mixed effects models, the frequency of citrus consumption was highly predictive of elevated Pro-B (Table 10).

## 4. Discussion

This study aimed to inform the use of a urinary biomarker of citrus intake in pregnant women. Here, the magnitude of WIV in urinary Pro-B concentrations, measured by ^1^H-NMR spectroscopy in repeated spot or 24-h urine specimens, was quantified and potential sources of temporal variation during pregnancy (i.e., seasonal, gestational, and residual random variation) were identified. In parsing out sources of this variance, citrus season (December–May) was a significant predictor of greater urinary Pro-B concentrations, while gestational age was inversely associated with non-normalized Pro-B concentrations. In linear mixed effects models, a high degree of WIV as a percentage of the total variance in urinary Pro-B was discovered (≥69%) regardless of the normalization method, indicating that multiple samples per participant are likely needed when using this biomarker to assess usual citrus intake or Pro-B exposure. Finally, moderate correlations were found between usual citrus intake reported on a semi-quantitative FFQ and single or averaged repeated urinary Pro-B measurements, with stronger correlations found with repeated measures.

Limited literature exists on the WIV of urinary Pro-B in free-living populations. Wang et al. recently reported intraclass correlation coefficients (ICCs) of relative abundance of candidate dietary biomarkers, including Pro-B, measured in two repeat samples collected 6 months apart, and found a slightly lower proportion of variance from WIV than in the current study (0.56 vs. ≥0.69) [48]. However, in addition to studying a different population, the methods used in that study differed from our study in several aspects; in particular, in the Wang et al. study, all specimens were from 24-h urine collections, relative abundance rather than absolute concentrations were measured, and values were normalized to osmolality [48]. Here, we report relatively high proportions of variance from WIV in 1 to 5 urine specimens, 85% of which were spot collections, collected throughout pregnancy. A higher degree of WIV was found among the spot urines compared to the combined sample; thus, more repeated samples may be needed for studies collecting spot urines compared to 24-h urine specimens.

A number of potential sources of variation may explain the high level of WIV found in urinary Pro-B. First, given the short- to medium-term nature of this biomarker in urine and its strong postprandial response to the intake of citrus products [37,39,56,57], large fluctuations in Pro-B concentration are likely largely attributable to true variation in intake. However, the portion of variation reflective of true intake is a function not only of the quantity consumed, but also of the time since consumption (especially in spot urine collections) and, potentially, the type of citrus product eaten. Considerable variation in Pro-B content has been reported among citrus fruit varieties [37,39] and forms (e.g., 1316 ± 72 mg/L vs. 761 ± 89 mg/L vs. 251 ± 153 mg/L in orange juice from concentrate, orange, and lemon, respectively [39]); thus, the specific products consumed may be influential on measured Pro-B concentrations. Apart from variations in the amount and type of citrus consumed, inter-individual differences or intra-individual changes in digestion, absorption, and metabolism of consumed foods and their components may influence the Pro-B concentration in a given urine sample. Although a large amount of ingested Pro-B is excreted unchanged, biotransformation products including sulfate and monoglucuronide derivatives have been identified in urine after consumption [57], and it is possible these processes could be influenced by person-specific factors. More research is needed to determine the extent to which both person-specific and temporal factors may affect the metabolism and subsequent excretion of Pro-B.

Citrus season at the time of sample collection was found to be a significant predictor of Pro-B levels above 30 µmol/mmol creatinine and of urinary Pro-B concentration in continuous data analyses. In line with these results, a cohort study of women living in and around Grand Forks, North Dakota found higher reported citrus consumption during the winter and spring seasons [71]. Seasonal variation was also observed in serum levels of β-cryptoxanthin, a carotenoid found in orange-flesh fruits and vegetables, among adults in a rural area of Japan, with related seasonal changes in reported Satsuma mandarin consumption [72]. Evidence of seasonal variation in dietary patterns and nutrient intakes has been well established in rural settings where local agriculture is likely to influence food availability and/or dietary consumption patterns, or where wild foods are available seasonally [13,16,73,74]. In contrast, studies conducted in industrialized settings have yielded mixed results in demonstrating seasonal variation in dietary intakes of energy, nutrients, and/or food groups [75,76], which appears to be declining over time [76], perhaps due to increased globalization of food markets allowing for year-round importation of seasonal products [77]. However, as shown in a U.S. cohort, the consumption of specific fruits and vegetables may vary seasonally with no or incongruent fluctuations in overall food groups or nutrients [71]. It may be particularly plausible that in locations of high agricultural productivity, such as the current study area, consumption of seasonal produce items grown in the area (e.g., citrus) could vary by season due to increased availability from local markets and residential fruit trees. Nevertheless, in the current analyses, season explained only a small proportion (3%) of the variation in urinary Pro-B, and its relationship with elevated Pro-B in logistic regression analyses did not reach statistical significance for non-normalized data (Table 5). The latter result may point to the creatinine-normalized cutoff as a better indicator of recent citrus consumption. To summarize, these results indicate that even in industrialized settings, agricultural season may have some influence on dietary intakes of particular foods, and that these changes are detectable in a biomarker of citrus consumption in pregnant women living in Northern California. These results are likely context-specific; therefore, dietary surveys and epidemiological studies assessing diet should consider the local context and be designed to account for this potential source of variation.

A small, statistically significant negative association was found between gestational age and non-normalized urinary Pro-B concentration, suggesting a slight reduction in Pro-B over the course of gestation. In contrast to citrus season, which is assumed to influence Pro-B levels largely through true changes in diet, gestational age could potentially be associated with Pro-B levels either due to dietary changes associated with pregnancy progression or through changes in physiology affecting digestion, metabolism, or urinary excretion dynamics. With regard to diet, some evidence suggests variation in dietary intake of certain food groups [19,78] and nutrients [78,79] throughout pregnancy. For example, a study of Canadian women observed decreases in the fruit and vegetable sub-score of the Canadian Healthy Eating Index across pregnancy trimesters [19]. This study also found decreases across trimesters in reported nausea (a well-known phenomenon), food cravings, and food aversions [19], conditions that can influence dietary consumption behavior during pregnancy [20,78]. In particular, in a study assessing self-reported dietary changes in pregnancy and reasons thereof, craving was found to be the most commonly named reason for increasing intake of foods, with fruit among the top listed foods that were increased for this reason [20]. Thus, changes in fruit consumption during pregnancy is plausible, although consistent trends across gestation may not be expected. Accordingly, we observed no association between gestational age and Pro-B above the defined thresholds, a likely indicator of recent citrus consumption, in our logistic regression analyses.

With respect to potential biological effects of gestation on urinary biomarker levels, changes in kidney anatomy and function occurring during pregnancy, e.g., increased glomerular filtration rate and kidney volume and decreased reabsorption of certain substances such as glucose (reviewed in [17]), could conceivably influence Pro-B concentrations in spot and 24-h urine samples. However, although several studies point to Pro-B being minimally metabolized and rapidly excreted [37,39,56,80], an in-depth understanding of the renal mechanisms by which Pro-B is excreted in mammals appears to be lacking, pointing to the need for more comprehensive pharmacokinetic research [81]. Given this research gap, the potential effects of changes in kidney function on urinary Pro-B concentration, as well as the appropriateness of adjustment for hydration status by creatinine normalization [18,82], remain unclear. Finally, it is important to consider that the beta coefficients reported here indicated only a small association between non-normalized Pro-B and gestational age and thus may not translate into meaningful biological or dietary changes. Based on the inconsistent results regarding this association for non-normalized vs. creatinine-normalized Pro-B levels and the unavailability of acute dietary intake data in most participants, additional research is needed to investigate whether gestational age augments the quantitative relationship between citrus consumption and Pro-B levels and whether it should be accounted for when using Pro-B as a dietary biomarker during pregnancy. Nevertheless, given the potential for dietary changes throughout pregnancy and the existence of critical windows for nutritional effects on fetal development [83,84,85,86,87], biomarker measurements taken during multiple trimesters or at hypothesis-specific time points during pregnancy will likely remain relevant for dietary surveys and epidemiological studies.

Our study complements and extends upon previously reported data on the correlation between short- to medium-term citrus intake and urinary Pro-B levels [39,55,58], as well as studies relating relative abundance of Pro-B to long-term reported intake [45,46,48,57]. First, we applied a previously reported threshold for creatinine-normalized urinary Pro-B relevant to recent citrus consumption [56] and found it performed moderately well in a subset of samples for which 24HDR data were available, with similar results for a cutoff derived for non-normalized Pro-B. Given that for some misclassified samples it was discovered that items potentially containing citrus were reportedly consumed (although their citrus content could not be confirmed), our finding of 82.6% and 78.3% agreement between the biomarker cutoffs and reported intake is likely an underestimate. Second, by using repeated measures of quantified Pro-B in urine in relation to a measure of usual citrus intake during pregnancy, we have shown that reported usual citrus consumption, both in terms of frequency of consumption and average daily servings, is highly predictive of elevated urinary Pro-B in short-term urine specimens and moderately correlated with urinary Pro-B concentration averaged from repeated measurements, respectively. The strength of this correlation is likely impacted by several, uncorrelated sources of measurement error in the biomarker and dietary recall instrument. For example, while a FFQ is designed to directly capture usual intake within a specified period of time, substantial recall bias may occur due to social desirability bias, imperfect recollection, and other person-specific factors [88,89]. Furthermore, although urinary Pro-B is not subject to recall bias, this measure is more akin to a 24HDR in that it reflects more of the random day-to-day variation in dietary intake, which introduces error when used as a measure of usual intake. In partial support of this, the correlation coefficients observed in this study suggest stronger correlations when 2 vs. 1, and possibly 3 vs. 2, measures per individual are used (Figure 2 and Figures S3 and S4, Supplemental Materials). However, more research is needed to confirm whether the collection of >2 specimens leads to stronger correlations, given the small number of individuals with 3 or more repeats available in this study. Nevertheless, based on the estimated variance components, the number of required samples to estimate usual Pro-B level or rank individuals with a desirable degree of accuracy was higher than the number commonly collected or likely to be considered feasible to collect in large population studies (Table 8); thus, modeling strategies to account for random day-to-day variation and other measurement error similar to those applied to 24HDR data [22,23,90] may be preferable to averaged repeats when analyzing urinary Pro-B data to assess usual citrus exposure for population distributions or epidemiological studies. A second source of biomarker error may derive from the consumption of non-citrus sources of Pro-B [37,39,62,63,64,65,66]. The importance of this source of error is likely population-specific as it depends on the consumption of a small number of specific food items with substantial Pro-B levels (e.g., gorgonzola cheese). In our study population, such items were not reportedly consumed among the subsample of participants for which 24HDR data were available, which in part could have been influenced by dietary safety recommendations during pregnancy advising against consumption of certain seafood and soft cheeses [91]. Thirdly, the relationship between dietary intake and biomarkers is influenced by the digestion, absorption, metabolism, and excretion of the biomarker (or its dietary precursors), which could vary across individuals. To elucidate these factors for Pro-B in the context of citrus consumption, existing pharmacokinetic studies, which have focused largely on orange juice consumption [37,39,56], should be expanded to investigate the aforementioned factors and include a range of commonly consumed citrus juices and fruits.

Several limitations should be considered in interpreting the results. First, the use of spot urine specimens collected at the same time of day (first-morning void) may not be ideal for representing citrus consumption over the course of a whole day. Some previous research has observed that most Pro-B excretion occurs in the first 14 h following orange juice consumption [39]; thus, collecting spot urine specimens only at the first-morning void may systematically under-measure citrus consumed in the morning, since this timing may not capture the previous or current morning’s consumption. A solution to this problem might be to alternate the timing of spot urine collections at different times during the day to ensure the representativeness of morning, midday, and evening consumption among individuals. Second, while taking a ^1^H-NMR metabolomics approach to measuring Pro-B offers several advantages, including the ability to obtain absolute quantification of multiple compounds using a single experiment, this method is limited in its sensitivity compared to other approaches, such as mass spectrometry, which can detect concentrations in the nanomolar range. The resulting uncertainty in the quantification of Pro-B present in low concentrations, which occurred in a substantial proportion of samples in the present study, may have influenced the variance component estimates reported here. However, we also present a second approach to data analysis that avoids this uncertainty by considering a concentration threshold likely indicative of recent citrus intake. Employing a two-part, probability × amount model, as has previously been conducted in dietary recall analysis to account for the often episodic nature of food consumption [22,25] may allow researchers to maintain the efficiency of measuring multiple biomarkers simultaneously (as opposed to opting for a more sensitive method) while reducing this uncertainty in the quantitative data. A third potential limitation is the unknown effects of long storage times and up to three freeze-thaw cycles on the stability of Pro-B in urine samples. Although no previous literature was found to address these questions in the case of urine, one study reported high percent recoveries (means of 94–100%) of Pro-B in rat plasma after three freeze-thaw cycles or 30 days of storage at −20 °C [92]. In the present study, no clear pattern in urinary Pro-B was observed by the number of freeze-thaw cycles or storage time. Measurement error in the estimation of gestational age is common and may have been present in this study; such error can introduce bias into measured associations under some circumstances [93]. Finally, it should be noted that the FFQ form used in this study was not specifically validated for use in pregnant women to cover food intake during pregnancy; instead, the defined recall period was modified to cover the first or second half of pregnancy by way of written and/or oral instructions given to participants at the time of form administration (for question wording, see Table S1, Supplemental Materials). This discrepancy might have introduced additional error into participants’ estimation of food intake for the period of interest and influenced the biomarker-FFQ correlation.

This study also has several strengths. We provide for the first time a quantitative analysis of the within- and between-individual variance components of urinary Pro-B concentration in pregnant women, an understudied population in terms of research on this biomarker. Analyzing up to 5 specimens per person covering all trimesters of pregnancy and seasons allowed us to differentiate random day-to-day variation from two potential sources of systematic WIV in biomarker levels—gestational age and citrus production season—and to test the effect of the number of samples per individual on the correlation with reported usual intake. Collecting >2 repeated measures also may have allowed for better variance component estimation than collecting fewer repeats and/or collecting repeats on a smaller subset of participants, although additional research is needed to determine the ideal timing between and the number of repeated measurements. Another strength was the inclusion of data from spot urine collections along with 24-h specimens, which allowed for the assessment of whether the collection method contributed to variation in the biomarker and for the observation of a higher degree of WIV in Pro-B among spot urine samples. In the context of large epidemiological studies, using spot urine samples carries the advantage of having a smaller participant burden relative to 24-h collections; thus, the results presented here can inform future studies aiming to use this measure to assess usual citrus intake in free-living populations.

Some additional research is warranted before relying on Pro-B as a quantitative measure of citrus intake, whether acute or usual, for the purposes of replacing, validating, or correcting for measurement errors in dietary recall instruments. Notably, recently developed calibration equations that successfully predicted mean total citrus intake over 4 consecutive days based on a single first void urine specimen in an Irish population [55,58] are promising and should be validated in other populations with varying dietary habits and genetic backgrounds, as well as in pregnant populations. From this angle, further defining the temporal limits of the predictive ability of a single urinary measurement would be of value (i.e., how many days’ intake can be quantitatively predicted from a single measurement?). While our study provides useful data towards the goal of quantifying the relationship between usual citrus consumption and urinary Pro-B concentrations in repeated urine specimens, given that long-term usual intake is not directly observable as a comparative measure, additional studies to understand the short-term excretion kinetics and percent recovery of dietary Pro-B from a variety of citrus products in diverse populations may further inform the development of (1) mathematical models for quantitative citrus intake prediction, and (2) urine sampling protocols to optimize the number and timing of repeated measures for estimating citrus intake for a given time period. (For example, one approach may involve the collection of 2 or 3 spot urine specimens collected throughout the day to better capture spikes in Pro-B levels and differentiate between recent consumption of a small portion and consumption of a larger portion several hours ago.) Importantly, the aforementioned research gaps apply to pregnant women as well as other subpopulations of interest for nutrition monitoring and epidemiology (e.g., non-pregnant adults, children, etc.).

## 5. Conclusions

A high degree of WIV in urinary Pro-B concentrations was observed among pregnant women in a Northern Californian cohort. In this area of high local citrus production, citrus season accounted for a small, statistically significant portion of the total variance in Pro-B (3%). The contribution of gestational age to WIV of Pro-B was less clear, as a weak negative association was observed with non-normalized Pro-B only. Single or averaged repeated urinary Pro-B concentrations (1–5 samples per person) were moderately correlated with usual citrus intake as reported by FFQ. Collecting ≥2 urine specimens per individual may strengthen this correlation and more closely represent usual intake, but more research with a larger sample size of individuals with ≥3 repeated measurements is needed to confirm the additional benefit of a greater number of repeats. Usual intake/exposure models based on the decomposition of the total variance into WIV and BIV are likely necessary to represent usual citrus intake/Pro-B exposure more accurately for the purpose of characterizing population distributions or epidemiological associations. NMR spectroscopy is a convenient method for quantifying Pro-B in urine, particularly if a simultaneous measurement of multiple biomarkers is desired, but may require the use of relevant cutoffs and 2-part (probability × amount) models for accurate analysis given the limited sensitivity of the method. Given the often-episodic nature of citrus fruit consumption and the high degree of WIV in biomarker values reported here, multiple urine specimens per individual should be collected in dietary studies aiming to quantify usual citrus intake and/or Pro-B exposure.

## Figures and Tables

**Table 1:** Urine specimen characteristics ^1^, by type of specimen.

	Spot Urines (*n* = 216)	24-h Urines (*n* = 39)	Total (*n* = 255)
Trimester of pregnancy			
1st	18 (8.3%)	1 (2.6%)	19 (7.5%)
2nd	102 (47.2%)	9 (23.1%)	111 (43.5%)
3rd	96 (44.4%)	29 (74.4%)	125 (49.0%)
Year of specimen collection			
2007	20 (9.3%)	0 (0.0%)	20 (7.8%)
2008	30 (13.9%)	6 (15.4%)	36 (14.1%)
2009	38 (17.6%)	8 (20.5%)	46 (18.0%)
2010	30 (13.9%)	5 (12.8%)	35 (13.7%)
2011	20 (9.3%)	3 (7.7%)	23 (9.0%)
2012	21 (9.7%)	6 (15.4%)	27 (10.6%)
2013	30 (13.9%)	4 (10.3%)	34 (13.3%)
2014	27 (12.5%)	7 (17.9%)	34 (13.3%)
Creatinine concentration, µM			
Median (Q1,Q3)	5597 (3818, 8318)	5083 (3663, 6512)	5464 (3795, 7976)
Sample pH			
Median (Q1,Q3)	6.87 (6.83, 6.93)	6.85 (6.82, 6.88)	6.87 (6.83, 6.92)

^1^ Categorical data are presented as count (%), continuous variables as median (Q1, Q3).

**Table 2:** Urinary proline betaine concentrations ^1^ by specimen and participant characteristics.

	*N*	Proline Betaine, µM	Proline Betaine, µmol/mmol Creatinine
All samples	255	73.1 (22.2, 258.8) ^2^	12.1 (4.5, 49.2)
Specimen type			
Spot	216	65.7 (22.4, 247.1)	11.6 (4.5, 44.9)
24-h	39	83.9 (19.9, 413.9)	15.9 (4.7, 68.3)
Specimen collection year			
2007–2010	137	65.9 (20.3, 259.8)	14.3 (4.6, 54.2)
2011–2014	118	76.2 (24.3, 240.2)	11.1 (4.4, 43.3)
No. of previous thaws			
0	31	60.7 (24.5, 96.6)	7.9 (4.7, 25.8)
1	217	78.3 (22.4, 302.9)	15.7 (4.6, 52.2)
2–3	7	30.7 (13.7, 55.4)	5.9 (2.7, 11.5)
Trimester of pregnancy			
1st	19	81.6 (43.8, 223.8)	15.7 (5.3, 43.2)
2nd	111	97.2 (24.2, 328.8)	19.1 (6.2, 52.6)
3rd	125	58.8 (19.1, 191.7)	9.8 (2.9, 43.1)
Maternal age at delivery			
<35 years	122	70.1 (23.9, 369.2)	10.8 (3.4, 54.2)
≥35 years	133	73.1 (21.4, 217.9)	15.7 (5.3, 43.7)
Gestational diabetes			
Yes	51	55.2 (18.1, 199.2)	11.7 (5.2, 35.7)
No	202	74.3 (22.6, 269.2)	12.1 (4.4, 49.9)

^1^ Proline betaine concentrations reflect values after imputing observations of non-detectable proline betaine with 0.5*LOD (approach 1). ^2^ All values are expressed as median (Q1, Q3).

**Table 3:** Estimated mean urinary concentration of proline betaine accounting for repeated measures, by citrus season.

	*Citrus Season* (*n* = 128 ^1^)		*Non-Citrus Season* (*n* = 127 ^1^)				
Units	Geometric Mean ^2^	95% CI ^2^	Geometric Mean ^2^	95% CI ^2^	Difference ^2,3^	95% CI ^2,3^	*p*-Value
µmol/L	96.5	(71.5, 130.3)	57.70	(42.5, 78.4)	1.72	(1.2, 2.5)	0.005
µmol/mmol creatinine	17.3	(12.9, 23.1)	10.58	(7.8, 14.3)	1.68	(1.2, 2.4)	0.005

^1^ Number of total specimens. ^2^ Exponentiated statistic from random effects model analyzed with log values accounting for clustering by subject. ^3^ Exponentiated statistic from the mixed effects model: log(proline betaine)~(citrus season) + (1|Subject ID), where (1|Subject ID) is the random intercept for the effect of clustering by subject. The difference represents the ratio of geometric means of the citrus season group to the non-citrus season group.

**Table 4:** Temporal predictors of urinary proline betaine concentration ^1^.

	Unadjusted			Adjusted	
Predictor	Beta Coefficient (95% CI)	*p* Value	Marginal R-Squared ^2^	Beta Coefficient (95% CI)	*p* Value
Citrus season (December–May)					
Non-normalized Pro-B	0.54 (0.17, 0.91)	0.005 **	0.03	0.52 (0.16, 0.88) ^3^	0.005 **
Creatinine-normalized Pro-B	0.52 (0.16, 0.88)	0.005 **	0.03	0.51 (0.15, 0.88) ^4^	0.006 **
Gestational age, months					
Non-normalized Pro-B	−0.11 (−0.20, −0.017)	0.022 *	0.02	−0.093 (−0.18, −0.0038) ^5^	0.042 *
Creatinine-normalized Pro-B	−0.089 (−0.18, 0.0025)	0.058	0.01	−0.070 (−0.16, 0.021) ^6^	0.133

^1^ Continuous variables with the units µmol/L and µmol/mmol creatinine for non-normalized and creatinine-normalized data, respectively. Data were ln-transformed before analysis. ^2^ Proportion of variance explained by the predictor. ^3^ Adjusted for log creatinine and gestational age at the time of sample collection. ^4^ Adjusted for maternal metabolic conditions and gestational age at the time of sample collection. The analytical sample size is 249 due to missing metabolic conditions data. ^5^ Adjusted for log creatinine and citrus season at the time of sample collection. ^6^ Adjusted for maternal metabolic conditions and citrus season at the time of sample collection. The analytical sample size is 249 due to missing metabolic conditions data. * *p* < 0.05; ** *p* < 0.01. Abbreviations: Pro-B, proline betaine.

**Table 5:** Relationship between elevated urinary proline betaine concentration and citrus season.

	Overall Proportion of Samples with Elevated Pro-B			Association between Elevated Pro-B and Citrus Season	
Threshold for Elevated Pro-B	All Samples (*n* = 255)	Non-Citrus Season ^1^ (*n* = 127)	Citrus Season ^2^ (*n* = 128)	Adjusted odds Ratio (95% CI) ^3^	*p* Value
>100 µmol/L	0.42	0.36	0.48	1.70 (0.92, 3.14)	0.09
>30 µmol/mmol creatinine	0.35	0.29	0.41	2.06 (1.03, 4.11)	0.04 *

^1^ Months of June through November. ^2^ Months of December through May. ^3^ Mixed effects logistic regression results. The non-normalized data analysis is adjusted for log urinary creatinine, and the creatinine-normalized analysis is adjusted for length of time in storage before analysis. * *p* < 0.05.

**Table 6:** Model descriptions, variance components, and variance ratios of urinary proline betaine, all data.

Biomarker	Model	Covariates	No. of Observations	Mean of Ln-Transformed Biomarker Concentration	WIV	BIV	WIV:BIV	WIV to Total Variance Ratio
Urinary pPro-B, µM ^1^	Unadjusted	-	255	4.31	1.82	0.65	2.82	0.74
Urinary Pro-B, µM ^1^	Adjusted	Citrus season ^2^, gestational age, urinary creatinine ^1^	255	4.31	1.57	0.68	2.31	0.70
Urinary Pro-B, µmol/mmol creatinine ^1^	Unadjusted	-	255	2.61	1.66	0.67	2.47	0.71
Urinary Pro-B, µmol/mmol creatinine ^1^	Adjusted	Citrus season, gestational age, metabolic conditions ^3^	249 ^4^	2.61	1.58	0.72	2.20	0.69

^1^ Ln-transformed. ^2^ Includes the months of December through May. ^3^ Five-category variables including (1) healthy weight with no metabolic conditions, (2) overweight with no metabolic conditions, (3) obesity with no other metabolic conditions, (4) hypertension/preeclampsia without diabetes mellitus, and (5) type 2 diabetes and/or gestational diabetes with or without hypertension/preeclampsia. ^4^ Sample sizes differ due to missing metabolic conditions data. Abbreviations: WIV, within-individual variance; BIV, between-individual variance.

**Table 7:** Model descriptions, variance components, and variance ratios of urinary proline betaine concentrations, including data above relevant thresholds ^1^.

Biomarker	Model	Covariates	No. of Observations	Mean of ln-Transformed Biomarker Concentration	WIV	BIV	WIV:BIV	WIV to Total Variance Ratio
Urinary proline betaine, µM ^2^	Unadjusted	-	108	5.81	0.49	0.15	3.27	0.77
Urinary proline betaine, µM ^2^	Adjusted	Urinary creatinine ^2^	108	5.81	0.45	0.16	2.88	0.74
Urinary proline betaine, µmol/mmol creatinine ^2^	Unadjusted	-	89	4.31	0.18	0.18	0.99	0.50
Urinary proline betaine, µmol/mmol creatinine ^2^	Adjusted	Season ^3^, metabolic conditions ^4^	87 ^5^	4.31	0.16	0.19	0.86	0.46

^1^ >100 µM and >30 µmol/mmol creatinine for non-normalized and creatinine-normalized data, respectively. ^2^ Log-transformed. ^3^ Four seasons. ^4^ Five-category variable including (1) healthy weight with no metabolic conditions, (2) overweight with no metabolic conditions, (3) obesity with no other metabolic conditions, (4) hypertension/preeclampsia without diabetes mellitus, and (5) type 2 diabetes and/or gestational diabetes with or without hypertension/preeclampsia. ^5^ Differing sample size is due to missing metabolic conditions data. Abbreviations: WIV, within-individual variance; BIV, between-individual variance.

**Table 8:** Estimated number of samples required per individual to determine true usual urinary proline betaine and to rank individuals on usual proline betaine levels.

Purpose	Non-Normalized Pro-B ^1^	Creatinine-Normalized Pro-B ^2^
True usual Pro-B ± 5% ^3^	151	255
True usual Pro-B ± 10% ^3^	38	64
True usual Pro-B ± 15% ^3^	17	28
True usual Pro-B ± 20% ^3^	9	16
Rank individuals with *r*^2^ of ≥0.9	25	22
Rank individuals with *r*^2^ of ≥0.8	11	10
Rank individuals with *r*^2^ of ≥0.7	7	6
Rank individuals with *r*^2^ of ≥0.5	3	2

^1^ The within-individual coefficient of variation is 31.35%. ^2^ The within-individual coefficient of variation is 40.75%. ^3^ *Z_α_* = 1.96.

**Table 9:** Correlations between averaged urinary proline betaine concentrations and FFQ-reported usual citrus intake (servings/day), in all samples.

Biomarker	Data Transformation	Correlation Coefficient (95% CI)	*p* Value
Urinary proline betaine, µM	Original	0.40 ^1^	<0.0001
Urinary proline betaine, µM	Log_e_ ^2^	0.45 (0.29, 0.59) ^3^	<0.0001
Urinary proline betaine, µmol/mmol creatinine	Original	0.42 ^1^	<0.0001
Urinary proline betaine, µmol/mmol creatinine	Log_e_ ^2^	0.48 (0.32, 0.61) ^3^	<0.0001

^1^ Spearman correlation. ^2^ Transformation applied to both FFQ and proline betaine variables. ^3^ Pearson correlation. Abbreviations: FFQ, food frequency questionnaire.

**Table 10:** Association between elevated urinary proline betaine and FFQ-reported frequency of citrus fruit or juice consumption, accounting for repeated measures.

		Unadjusted			Adjusted ^1^	
Threshold for Elevated Pro-B	Odds Ratio (95% CI)	*p*-Value	R-Squared ^2^	Odds Ratio (95% CI)	*p* Value	R-Squared ^2^
>100 µmol/L	20.1 (5.4, 75.3)	<0.00001	0.11	22.8 (6.6, 79.3)	<0.000001	0.19
>30 µmol/mmol creatinine	20.5 (4.7, 89.7)	<0.0001	0.10	20.7 (4.6, 92.8)	<0.0001	0.12

^1^ Non-normalized data analysis is adjusted for maternal height, log-transformed urinary creatinine, and citrus season. Creatinine-normalized data analysis is adjusted for citrus season. ^2^ Calculated by the delta method. Marginal R-squared describes the variance explained by the fixed effect(s) in the model. Abbreviations: FFQ, food frequency questionnaire; Pro-B, proline betaine.

## Data Availability

The data presented in this study are available on request from the corresponding author. The data are not publicly available due to the need to protect participant privacy.

## Supplementary Materials

The following supporting information can be downloaded at: https://www.mdpi.com/article/10.3390/metabo13080904/s1, Figure S1: Distribution of gestational age at the time of urine specimen collections; Figure S2: Scatter plots of log urinary proline betaine concentration (µmol/mmol creatinine) by log reported usual citrus intake (servings/day), comparing single sample data with averaged repeated measures. R represents Pearson correlation coefficient. Blue lines are derived from linear regression; Figure S3: Scatter plots of urinary proline betaine concentration (µmol/mmol creatinine) by reported usual citrus intake (servings/day) including varying numbers of repeated measures, for a subset of individuals with at least 3 samples available. R represents Spearman’s rank correlation coefficient. Blue lines are derived from linear regression and are included to visualize trends in the data; Figure S4: Scatter plots of urinary proline betaine concentration (µmol/mmol creatinine) by reported usual citrus intake (servings/day) including varying numbers of repeated measures, for a subset of individuals with at least 4 samples available. R represents Spearman’s rank correlation coefficient. Blue lines are derived from linear regression and are included to visualize trends in the data. Table S1: FFQ variable descriptions and assumptions used to calculate daily citrus consumption; Table S2: Characteristics of MARBLES study participants (*n* = 107) with NMR-analyzed urine specimens collected during pregnancy; Table S3: Profiling of urinary NMR signals for proline betaine identification and quantification; Table S4: Frequency and proportion of urine samples with elevated proline betaine (>100 µM), by the number of samples; Table S5: Model descriptions, variance components, and variance ratios of urinary proline betaine concentrations, spot urines only; Table S6: Associations between proline betaine concentration and temporal predictors, only including data above thresholds likely indicative of citrus consumption.

## References

[1] Willett W. (2013). Nutritional Epidemiology.

[2] Krebs-Smith S.M., Graubard B.I., Kahle L.L., Subar A.F., Cleveland L.E., Ballard-Barbash R. (2000). Low Energy Reporters vs Others: A Comparison of Reported Food Intakes. Eur. J. Clin. Nutr..

[3] Kipnis V., Subar A.F., Midthune D., Freedman L.S., Ballard-Barbash R., Troiano R.P., Bingham S., Schoeller D.A., Schatzkin A., Carroll R.J. (2003). Structure of Dietary Measurement Error: Results of the OPEN Biomarker Study. Am. J. Epidemiol..

[4] Kupper L.L. (1984). Effects of the Use of Unreliable Surrogate Variables on the Validity of Epidemiologic Research Studies. Am. J. Epidemiol..

[5] Kipnis V., Midthune D., Freedman L.S., Bingham S., Schatzkin A., Subar A., Carroll R.J. (2001). Empirical Evidence of Correlated Biases in Dietary Assessment Instruments and Its Implications. Am. J. Epidemiol..

[6] Hedrick V.E., Dietrich A.M., Estabrooks P.A., Savla J., Serrano E., Davy B.M. (2012). Dietary Biomarkers: Advances, Limitations and Future Directions. Nutr. J..

[7] Picó C., Serra F., Rodríguez A.M., Keijer J., Palou A. (2019). Biomarkers of Nutrition and Health: New Tools for New Approaches. Nutrients.

[8] Playdon M.C., Moore S.C., Derkach A., Reedy J., Subar A.F., Sampson J.N., Albanes D., Gu F., Kontto J., Lassale C. (2017). Identifying Biomarkers of Dietary Patterns by Using Metabolomics. Am. J. Clin. Nutr..

[9] Brennan L. (2017). The Nutritional Metabolomics Crossroads: How to Ensure Success for Dietary Biomarkers. Am. J. Clin. Nutr..

[10] Tasevska N., Midthune D., Potischman N., Subar A.F., Cross A.J., Bingham S.A., Schatzkin A., Kipnis V. (2011). Use of the Predictive Sugars Biomarker to Evaluate Self-Reported Total Sugars Intake in the Observing Protein and Energy Nutrition (OPEN) Study. Cancer Epidemiol. Biomark. Prev..

[11] Freedman L.S., Midthune D., Carroll R.J., Tasevska N., Schatzkin A., Mares J., Tinker L., Potischman N., Kipnis V. (2011). Using Regression Calibration Equations That Combine Self-Reported Intake and Biomarker Measures to Obtain Unbiased Estimates and More Powerful Tests of Dietary Associations. Am. J. Epidemiol..

[12] Tooze J.A., Grunwald G.K., Jones R.H. (2002). Analysis of Repeated Measures Data with Clumping at Zero. Stat. Methods Med. Res..

[13] Caswell B.L., Talegawkar S.A., Siamusantu W., West K.P., Palmer A.C. (2018). A 10-Food Group Dietary Diversity Score Outperforms a 7-Food Group Score in Characterizing Seasonal Variability and Micronutrient Adequacy in Rural Zambian Children. J. Nutr..

[14] Arsenault J.E., Nikiema L., Allemand P., Ayassou K.A., Lanou H., Moursi M., De Moura F.F., Martin-Prevel Y. (2014). Seasonal Differences in Food and Nutrient Intakes among Young Children and Their Mothers in Rural Burkina Faso. J. Nutr. Sci..

[15] Ferguson E.L., Gibson R.S., Opareobisaw C., Osei-Opare F., Lamba C., Ounpuu S. (1993). Seasonal Food Consumption Patterns and Dietary Diversity of Rural Preschool Ghanaian and Malawian Children. Ecol. Food Nutr..

[16] Caswell B.L., Talegawkar S.A., Siamusantu W., West K.P., Palmer A.C. (2020). Within-Person, between-Person and Seasonal Variance in Nutrient Intakes among 4- to 8-Year-Old Rural Zambian Children. Br. J. Nutr..

[17] Cheung K.L., Lafayette R.A. (2013). Renal Physiology of Pregnancy. Adv. Chronic Kidney Dis..

[18] MacPherson S., Arbuckle T.E., Fisher M. (2018). Adjusting Urinary Chemical Biomarkers for Hydration Status during Pregnancy. J. Expo. Sci. Environ. Epidemiol..

[19] Savard C., Lemieux S., Carbonneau É., Provencher V., Gagnon C., Robitaille J., Morisset A.S. (2019). Trimester-Specific Assessment of Diet Quality in a Sample of Canadian Pregnant Women. Int. J. Environ. Res. Public Health.

[20] Forbes L.E., Graham J.E., Berglund C., Bell R.C. (2018). Dietary Change during Pregnancy and Women’s Reasons for Change. Nutrients.

[21] McGowan C.A., McAuliffe F.M. (2014). Maternal Dietary Patterns and Associated Nutrient Intakes during Each Trimester of Pregnancy. Public Health Nutr..

[22] Tooze J.A., Midthune D., Dodd K.W., Freedman L.S., Krebs-Smith S.M., Subar A.F., Guenther P.M., Carroll R.J., Kipnis V. (2006). A New Statistical Method for Estimating the Usual Intake of Episodically Consumed Foods with Application to Their Distribution. J. Am. Diet. Assoc..

[23] Tooze J.A., Kipnis V., Buckman D.W., Carroll R.J., Freedman L.S., Guenther P.M., Krebs-Smith S.M., Subar A.F., Dodd K.W. (2010). A Mixed-Effects Model Approach for Estimating the Distribution of Usual Intake of Nutrients: The NCI Method. Stat. Med..

[24] Luo H., Dodd K.W., Arnold C.D., Engle-stone R. (2019). A New Statistical Method for Estimating Usual Intakes of Nearly-Daily Consumed Foods and Nutrients Through Use of Only One 24-Hour Dietary Recall. J. Nutr..

[25] Kipnis V., Midthune D., Buckman D.W., Dodd K.W., Guenther P.M., Krebs-Smith S.M., Subar A.F., Tooze J.A., Carroll R.J., Freedman L.S. (2009). Modeling Data with Excess Zeros and Measurement Error: Application to Evaluating Relationships between Episodically Consumed Foods and Health Outcomes. Biometrics.

[26] Intemann T., Mehlig K., De Henauw S., Siani A., Constantinou T., Moreno L.A., Molnár D., Veidebaum T., Pigeot I. (2019). SIMEX for Correction of Dietary Exposure Effects with Box-Cox Transformed Data. Biom. J..

[27] Turner T., Burri B.J. (2013). Potential Nutritional Benefits of Current Citrus Consumption. Agriculture.

[28] Farag M.A., Abib B., Ayad L., Khattab A.R. (2020). Sweet and Bitter Oranges: An Updated Comparative Review of Their Bioactives, Nutrition, Food Quality, Therapeutic Merits and Biowaste Valorization Practices. Food Chem..

[29] Landberg R., Sun Q., Rimm E.B., Cassidy A., Scalbert A., Mantzoros C.S., Hu F.B., van Dam R.M. (2011). Selected Dietary Flavonoids Are Associated with Markers of Inflammation and Endothelial Dysfunction in U.S. Women. J. Nutr..

[30] Cassidy A., Bertoia M., Chiuve S., Flint A., Forman J., Rimm E.B. (2016). Habitual Intake of Anthocyanins and Flavanones and Risk of Cardiovascular Disease in Men. Am. J. Clin. Nutr..

[31] Li L., Lyall G.K., Alberto Martinez-Blazquez J., Vallejo F., Tomas-Barberan F.A., Birch K.M., Boesch C. (2020). Blood Orange Juice Consumption Increases Flow-Mediated Dilation in Adults with Overweight and Obesity: A Randomized Controlled Trial. J. Nutr..

[32] Rendeiro C., Dong H., Saunders C., Harkness L., Blaze M., Hou Y., Belanger R.L., Corona G., Lovegrove J.A., Spencer J.P.E. (2016). Flavanone-Rich Citrus Beverages Counteract the Transient Decline in Postprandial Endothelial Function in Humans: A Randomised, Controlled, Double-Masked, Cross-over Intervention Study. Br. J. Nutr..

[33] Buscemi S., Rosafio G., Arcoleo G., Mattina A., Canino B., Montana M., Verga S., Rini G. (2012). Effects of Red Orange Juice Intake on Endothelial Function and Inflammatory Markers in Adult Subjects with Increased Cardiovascular Risk. Am. J. Clin. Nutr..

[34] McNamara A.E., Walton J., Flynn A., Nugent A.P., McNulty B.A., Brennan L. (2021). The Potential of Multi-Biomarker Panels in Nutrition Research: Total Fruit Intake as an Example. Front. Nutr..

[35] Cooper A.J.M., Sharp S.J., Luben R.N., Khaw K.T., Wareham N.J., Forouhi N.G. (2015). The Association between a Biomarker Score for Fruit and Vegetable Intake and Incident Type 2 Diabetes: The EPIC-Norfolk Study. Eur. J. Clin. Nutr..

[36] Mennen L.I., Sapinho D., Ito H., Bertrais S., Galan P., Hercberg S., Scalbert A. (2006). Urinary Flavonoids and Phenolic Acids as Biomarkers of Intake for Polyphenol-Rich Foods. Br. J. Nutr..

[37] Lang R., Lang T., Bader M., Beusch A., Schlagbauer V., Hofmann T. (2017). High-Throughput Quantitation of Proline Betaine in Foods and Suitability as a Valid Biomarker for Citrus Consumption. J. Agric. Food Chem..

[38] Cautela D., Vella F.M., Laratta B. (2019). The Effect of Processing Methods on Phytochemical Composition in Bergamot Juice. Foods.

[39] Heinzmann S.S., Brown I.J., Chan Q., Bictash M., Dumas M.E., Kochhar S., Stamler J., Holmes E., Elliott P., Nicholson J.K. (2010). Metabolic Profiling Strategy for Discovery of Nutritional Biomarkers: Proline Betaine as a Marker of Citrus Consumption. Am. J. Clin. Nutr..

[40] Cautela D., De Sio F., Balestrieri M.L., Casale R., Laratta B., Castaldo D., Pastore A., Servillo L., D’Onofrio N. (2020). Amino Acids, Betaines and Related Ammonium Compounds in Neapolitan Limmo, a Mediterranean Sweet Lime, Also Known as Lemoncetta Locrese. J. Sci. Food Agric..

[41] Mazzilli K.M., McClain K.M., Lipworth L., Playdon M.C., Sampson J.N., Clish C.B., Gerszten R.E., Freedman N.D., Moore S.C. (2020). Identification of 102 Correlations between Serum Metabolites and Habitual Diet in a Metabolomics Study of the Prostate, Lung, Colorectal, and Ovarian Cancer Trial. J. Nutr..

[42] de Souza R.J., Shanmuganathan M., Lamri A., Atkinson S.A., Becker A., Desai D., Gupta M., Mandhane P.J., Moraes T.J., Morrison K.M. (2020). Maternal Diet and the Serum Metabolome in Pregnancy: Robust Dietary Biomarkers Generalizable to a Multiethnic Birth Cohort. Curr. Dev. Nutr..

[43] Llorach R., Medina S., García-Viguera C., Zafrilla P., Abellán J., Jauregui O., Tomás-Barberán F.A., Gil-Izquierdo A., Andrés-Lacueva C. (2014). Discovery of Human Urinary Biomarkers of Aronia-Citrus Juice Intake by HPLC-q-TOF-Based Metabolomic Approach. Electrophoresis.

[44] Guertin K.A., Moore S.C., Sampson J.N., Huang W.Y., Xiao Q., Stolzenberg-Solomon R.Z., Sinha R., Cross A.J. (2014). Metabolomics in Nutritional Epidemiology: Identifying Metabolites Associated with Diet and Quantifying Their Potential to Uncover Diet-Disease Relations in Populations. Am. J. Clin. Nutr..

[45] Pujos-Guillot E., Hubert J., Martin J.F., Lyan B., Quintana M., Claude S., Chabanas B., Rothwell J.A., Bennetau-Pelissero C., Scalbert A. (2013). Mass Spectrometry-Based Metabolomics for the Discovery of Biomarkers of Fruit and Vegetable Intake: Citrus Fruit as a Case Study. J. Proteome Res..

[46] Lloyd A.J., Beckmann M., Haldar S., Seal C., Brandt K., Draper J. (2013). Data-Driven Strategy for the Discovery of Potential Urinary Biomarkers of Habitual Dietary Exposure. Am. J. Clin. Nutr..

[47] Lacalle-Bergeron L., Portoles T., Lopez F.J., Vicente Sacho J., Ortega-Azor C., Asensio E.M., Coltell O., Corella D. (2020). Ultra-Performance Liquid Chromatography-Ion Mobility Separation-Quadruple Time-of-Flight MS (UHPLC-IMS-QTOF MS) Metabolomics for Short-Term Biomarker Discovery of Orange Intake: A Randomized, Controlled Crossover Study. Nutrients.

[48] Wang Y., Hodge R.A., Stevens V.L., Hartman T.J., Mccullough M.L. (2021). Identification and Reproducibility of Urinary Metabolomic Biomarkers of Habitual Food Intake in a Cross-Sectional Analysis of the Cancer Prevention Study-3 Diet Assessment Sub-Study. Metabolites.

[49] Kim H., Hu E.A., Wong K.E., Yu B., Steffen L.M., Seidelmann S.B., Boerwinkle E., Coresh J., Rebholz C.M. (2021). Serum Metabolites Associated with Healthy Diets in African Americans and European Americans. J. Nutr..

[50] Lau C.H.E., Siskos A.P., Maitre L., Robinson O., Athersuch T.J., Want E.J., Urquiza J., Casas M., Vafeiadi M., Roumeliotaki T. (2018). Determinants of the Urinary and Serum Metabolome in Children from Six European Populations. BMC Med..

[51] Wellington N., Shanmuganathan M., De Souza R.J., Zulyniak M.A., Azab S., Bloomfield J., Mell A., Ly R., Desai D., Anand S.S. (2019). Metabolic Trajectories Following Contrasting Prudent and Western Diets from Food Provisions: Identifying Robust Biomarkers of Short-Term Changes in Habitual Diet. Nutrients.

[52] Almanza-Aguilera E., Urpi-Sarda M., Llorach R., Vázquez-Fresno R., Garcia-Aloy M., Carmona F., Sanchez A., Madrid-Gambin F., Estruch R., Corella D. (2017). Microbial Metabolites Are Associated with a High Adherence to a Mediterranean Dietary Pattern Using a 1H-NMR-Based Untargeted Metabolomics Approach. J. Nutr. Biochem..

[53] Kim H., Lichtenstein A.H., Wong K.E., Appel L.J., Coresh J., Rebholz C.M. (2020). Urine Metabolites Associated with the Dietary Approaches to Stop Hypertension (DASH) Diet: Results from the DASH-Sodium Trial. Mol. Nutr. Food Res..

[54] May D.H., Navarro S.L., Ruczinski I., Hogan J., Ogata Y., Schwarz Y., Levy L., Holzman T., McIntosh M.W., Lampe J.W. (2013). Metabolomic Profiling of Urine: Response to a Randomised, Controlled Feeding Study of Select Fruits and Vegetables, and Application to an Observational Study. Br. J. Nutr..

[55] Gibbons H., Michielsen C.J.R., Rundle M., Frost G., McNulty B.A., Nugent A.P., Walton J., Flynn A., Gibney M.J., Brennan L. (2017). Demonstration of the Utility of Biomarkers for Dietary Intake Assessment: Proline Betaine as an Example. Mol. Nutr. Food Res..

[56] Saenger T., Hübner F., Lindemann V., Ganswind K., Humpf H.U. (2020). Urinary Biomarkers for Orange Juice Consumption. Mol. Nutr. Food Res..

[57] Lloyd A.J., Beckmann M., Favé G., Mathers J.C., Draper J. (2011). Proline Betaine and Its Biotransformation Products in Fasting Urine Samples Are Potential Biomarkers of Habitual Citrus Fruit Consumption. Br. J. Nutr..

[58] D’Angelo S., Gormley I.C., McNulty B.A., Nugent A.P., Walton J., Flynn A., Brennan L. (2019). Combining Biomarker and Food Intake Data: Calibration Equations for Citrus Intake. Am. J. Clin. Nutr..

[59] Hertz-Picciotto I., Schmidt R.J., Walker C.K., Bennett D.H., Oliver M., Shedd-Wise K.M., LaSalle J.M., Giulivi C., Puschner B., Thomas J. (2018). A Prospective Study of Environmental Exposures and Early Biomarkers in Autism Spectrum Disorder: Design, Protocols, and Preliminary Data from the MARBLES Study. Environ. Health Perspect..

[60] Chawarska K., Shic F., Macari S., Campbell D.J., Brian J., Landa R., Hutman T., Nelson C.A., Ozonoff S., Tager-Flusberg H. (2014). 18-Month Predictors of Later Outcomes in Younger Siblings of Children with Autism Spectrum Disorder: A Baby Siblings Research Consortium Study. J. Am. Acad. Child Adolesc. Psychiatry.

[61] O’Sullivan A., Willoughby R.E., Mishchuk D., Alcarraz B., Cabezas-Sanchez C., Condori R.E., David D., Encarnacion R., Fatteh N., Fernandez J. (2013). Metabolomics of Cerebrospinal Fluid from Humans Treated for Rabies. J. Proteome Res..

[62] Servillo L., Giovane A., Casale R., Balestrieri M.L., Cautela D., Paolucci M., Siano F., Volpe M.G., Castaldo D. (2016). Betaines and Related Ammonium Compounds in Chestnut (*Castanea sativa* Mill.). Food Chem..

[63] Connor M.A., Stark J.B., Fritz J.C., Kohler G.O. (1973). Stachydrine: Content in Alfalfa and Biological Activity in Chicks. J. Agric. Food Chem..

[64] Al-Tamimi A., Khatib M., Pieraccini G., Mulinacci N. (2019). Quaternary Ammonium Compounds in Roots and Leaves of *Capparis spinosa* L. from Saudi Arabia and Italy: Investigation by HPLC-MS and 1H NMR. Nat. Prod. Res..

[65] Kärkkäinen O., Lankinen M.A., Vitale M., Jokkala J., Leppänen J., Koistinen V., Lehtonen M., Giacco R., Rosa-Sibakov N., Micard V. (2018). Diets Rich in Whole Grains Increase Betainized Compounds Associated with Glucose Metabolism. Am. J. Clin. Nutr..

[66] De Zwart F.J., Slow S., Payne R.J., Lever M., George P.M., Gerrard J.A., Chambers S.T. (2003). Glycine Betaine and Glycine Betaine Analogues in Common Foods. Food Chem..

[67] French C.D., Arsenault J.E., Arnold C.D., Haile D., Luo H., Dodd K.W., Vosti S.A., Slupsky C.M., Engle-Stone R., Engle-Stone R. (2020). Within-Person Variation in Nutrient Intakes across Populations and Settings: Implications for the Use of External Estimates in Modeling Usual Nutrient Intake Distributions. Adv. Nutr..

[68] Barton K. Package “MuMIn”.

[69] Beaton G.H., Milner J., Corey P., McGuire V., Cousins M., Stewart E., de Ramos M., Hewitt D., Grambsch P.V., Kassim N. (1979). Sources of Variance in 24-Hour Data: Implications for Nutrition Study Design and Interpretation. Am. J. Clin. Nutr..

[70] Ollberding N.J., Couch S.C., Woo J.G., Kalkwarf H.J. (2014). Within- and Between-Individual Variation in Nutrient Intake in Children and Adolescents. J. Acad. Nutr. Diet..

[71] Jahns L., Johnson L.A.K., Scheett A.J., Stote K.S., Raatz S.K., Subar A.F., Tande D. (2016). Measures of Diet Quality across Calendar and Winter Holiday Seasons among Midlife Women: A 1-Year Longitudinal Study Using the Automated Self-Administered 24-Hour Recall. J. Acad. Nutr. Diet..

[72] Sugiura M., Matsumoto H., Kato M., Ikoma Y., Yano M., Nagao A. (2004). Multiple Linear Regression Analysis of the Seasonal Changes in the Serum Concentration of β-Cryptoxanthin. J. Nutr. Sci. Vitaminol..

[73] Nyambose J., Koski K.G., Tucker K.L. (2002). High Intra/Interindividual Variance Ratios for Energy and Nutrient Intakes of Pregnant Women in Rural Malawi Show That Many Days Are Required to Estimate Usual Intake. J. Nutr..

[74] Golden C.D., Vaitla B., Ravaoliny L., Vonona M.A., Anjaranirina E.J.G., Randriamady H.J., Glahn R.P., Guth S.E., Fernald L.C.H., Myers S.S. (2019). Seasonal Trends of Nutrient Intake in Rainforest Communities of North-Eastern Madagascar. Public Health Nutr..

[75] Bernstein S., Zambell K., Amar M.J., Arango C., Kelley R.C., Miszewski S.G., Tryon S. (2017). Dietary Intake Patterns Are Consistent across Seasons in a Cohort of Healthy Adults in a Metropolitan Population. Physiol. Behav..

[76] Marti-Soler H., Guessous I., Gaspoz J.-M., Metcalf P., Deschamps V., Castetbon K., Malyutina S., Bobak M., Ruidavets J.-B., Bongard V. (2017). Seasonality of Nutrient Intake—An Analysis Including over 44,000 Participants in 4 Countries Reference Participants in 4 Countries. Clin. Nutr. ESPEN.

[77] Huang S.W. (2013). Imports Contribute to Year-Round Fresh Fruit Availability.

[78] Rifas-Shiman S.L., Rich-Edwards J.W., Willett W.C., Kleinman K.P., Oken E., Gillman M.W. (2006). Changes in Dietary Intake from the First to the Second Trimester of Pregnancy. Paediatr. Perinat. Epidemiol..

[79] Savard C., Lemieux S., Weisnagel S.J., Fontaine-Bisson B., Gagnon C., Robitaille J., Morisset A.S. (2018). Trimester-Specific Dietary Intakes in a Sample of French-Canadian Pregnant Women in Comparison with National Nutritional Guidelines. Nutrients.

[80] Lever M., Sizeland P.C.B., Bason L.M., Hayman C.M., Chambers S.T. (1994). Glycine Betaine and Proline Betaine in Human Blood and Urine. Biochim. Et Biophys. Acta-Gen. Subj..

[81] Cheng F., Zhou Y., Wang M., Guo C., Cao Z., Zhang R., Peng C. (2020). A Review of Pharmacological and Pharmacokinetic Properties of Stachydrine. Pharmacol. Res..

[82] Heavner D.L., Morgan W.T., Sears S.B., Richardson J.D., Byrd G.D., Ogden M.W. (2006). Effect of Creatinine and Specific Gravity Normalization Techniques on Xenobiotic Biomarkers in Smokers’ Spot and 24-h Urines. J. Pharm. Biomed. Anal..

[83] MRC Vitamin Study Research Group (1991). Prevention of Neural Tube Defects: Results of the Medical Research Council Vitamin Study. Lancet.

[84] Wald N.J., Hackshaw A.K., Stone R., Sourial N.A. (1996). Blood Folic Acid and Vitamin B12 in Relation to Neural Tube Defects. BJOG An. Int. J. Obstet. Gynaecol..

[85] Rodriguez-Diaz E., Pearce E.N. (2020). Iodine Status and Supplementation before, during, and after Pregnancy. Best Pract. Res. Clin. Endocrinol. Metab..

[86] Lyall K., Schmidt R.J., Hertz-Picciotto I. (2014). Maternal Lifestyle and Environmental Risk Factors for Autism Spectrum Disorders. Int. J. Epidemiol..

[87] Schmidt R.J., Hansen R.L., Hartiala J., Allayee H., Schmidt L.C., Tancredi D.J., Tassone F., Hertz-Picciotto I. (2011). Prenatal Vitamins, One-Carbon Metabolism Gene Variants, and Risk for Autism. Epidemiology.

[88] Gibson R. (2005). Measurement Errors in Dietary Assessment. Principles of Nutritional Assessment.

[89] Johansson L., Solvoll K., Bjørneboe G.E.A., Drevon C.A. (1998). Under- and Overreporting of Energy Intake Related to Weight Status and Lifestyle in a Nationwide Sample. Am. J. Clin. Nutr..

[90] Souverein O.W., Dekkers A.L., Geelen A., Haubrock J., de Vries J.H., Ocké M.C., Harttig U., Boeing H., van ‘t Veer P. (2011). Comparing Four Methods to Estimate Usual Intake Distributions. Eur. J. Clin. Nutr..

[91] U.S. Department of Agriculture, U.S. Department of Health and Human Services (2020). Dietary Guidelines for Americans, 2020–2025.

[92] Wen Y., Gong L., Wang L., Zhao N., Sun Q., Kamara M.O., Ma H., Meng F. (2019). Comparative Pharmacokinetics Study of Leonurine and Stachydrine in Normal Rats and Rats with Cold-Stagnation and Blood-Stasis Primary Dysmenorrhoea after the Administration of Leonurus Japonicus Houtt Electuary. J. Sep. Sci..

[93] Howards P.P., Hertz-Picciotto I., Weinberg C.R., Poole C. (2006). Misclassification of Gestational Age in the Study of Spontaneous Abortion. Am. J. Epidemiol..

