# Supplementary material for: Assessing Repeated Urinary Proline Betaine Measures as a Biomarker of Usual Citrus Intake during Pregnancy: Sources of Within-Person Variation and Correlation with Reported Intake
Source: Metabolites. 2023 Aug 2;13(8):904. doi: 10.3390/metabo13080904 (PMC10456298; doi:10.3390/metabo13080904)
Supplement: Supplementary file 1 [file metabolites-13-00904-s001.zip › metabolites-2506227-supplementary.pdf]

Supplemental Tables and Figures

Supplemental Tables

Table S1. FFQ variable descriptions and assumptions used to calculate daily citrus consumption.

<table>
<tr><th>FFQ question</th><th>Frequency response options</th><th>FFQ Item</th><th>Amount response options</th><th>Amount assumptions</th></tr>
<tr><td rowspan="3">How often do you eat the following foods all year round? Estimate your average for the whole year. [1]</td><td rowspan="3">“Never”
“A few times per year”
“Once per month”
“2-3 times per month”
“Once per week”
“2 times per week”
“3-4 times per week”
“5-6 times per week”
“Every day”</td><td>"Oranges or tangerines"</td><td>1/2 fruit
1 fruit
2 fruits</td><td rowspan="3">1 fruit = 1 cup equivalent [2]
“A little” = 1/4 cup equivalent
1 glass = 1 cup equivalent</td></tr>
<tr><td>"Grapefruit"</td><td>"A little"
1/2 grapefruit
1 grapefruit</td></tr>
<tr><td>"Real 100% orange juice or grapefruit juice."</td><td>1/2 glass
1 glass
2 glasses</td></tr>
</table>

[1] Clarifying instructions were given to each participant at the time of form administration indicating for which weeks the form should be completed, which corresponded to either the first or last 20 weeks of pregnancy.

[2] Approximate cup equivalent amounts for orange, grapefruit, and juice derived from MyPlate.gov (https://www.choosemyplate.gov/eathealthy/fruits, accessed February 10, 2021).

Table S2. Characteristics of MARBLES study participants (N=107) with NMR-analyzed urine specimens collected during pregnancy

	Mean (SD) or Count (%)
**Age at delivery, years [1]**	34.41 (5.09)
**Race/ethnicity**	
Non-Hispanic white	68 (63.6%)
Hispanic	21 (19.6%)
Black/African-American	3 (2.8%)
Asian	13 (12.1%)
Multiracial	2 (1.9%)
**Education**	
High school diploma or less	6 (5.6%)
Some college	22 (20.6%)
Associate’s degree or tech/vocational training	18 (16.8%)
Bachelor’s Degree	45 (42.1%)
Master’s, PhD, or other professional degree	16 (15.0%)
**Owns or rents home [2]**	
Rents	41 (38.7%)
Owns	65 (61.3%)
**Gestational diabetes [2,3]**	
No	86 (81.1%)
Yes	20 (18.9%)

[1] In the case of multiple pregnancies during the study (n=4), summary statistics reflect age at delivery of the first pregnancy.

[2] Percentages omit missing data (n=1).

[3] Gestational diabetes diagnoses were consistent across pregnancies among women with multiple pregnancies; women are counted once.

Table S3. Profiling of urinary NMR signals for proline betaine identification and quantification

Signal visibility	Count	Data Treatment
Could not see	57	Assigned as 0.5 × sample-specific detection limit
≥2 peaks visible with medium to high confidence	198	Used original value

Table S4. Frequency and proportion of urine samples with elevated proline betaine (>100 μM), by number of samples

Number of Samples per participant	Number of samples with elevated Pro-B from each individual	Proportion of an individual's samples with elevated Pro-B	Count (No. of participants)
1	0	0	5
1	1	1.0	7
2	0	0	25
2	1	0.5	17
2	2	1.0	15
3	0	0	7
3	1	0.33	9
3	2	0.67	7
3	3	1.0	2
4	0	0	2
4	1	0.25	1
4	2	0.5	5

4	3	0.75	2
4	4	1.0	1
5	2	0.4	2
**Total**			**107**

Abbreviation: Pro-B, proline betaine

Table S5. Model descriptions, variance components, and variance ratios of urinary proline betaine concentrations, spot urines only

Biomarker	Model	Covariates	WIV	BIV	WIV:BIV	WIV to total variance ratio
Urinary proline betaine, µM [1]	Unadjusted	-	2.02	0.34	5.95	0.86
Urinary proline betaine, µM [1]	Adjusted	Citrus season, gestational age, urinary creatinine [1]	1.63	0.49	3.33	0.77
Urinary proline betaine, µmol/mmol creatinine [1]	Unadjusted	-	1.82	0.43	4.26	0.81
Urinary proline betaine, µmol/mmol creatinine [1]	Adjusted	Citrus season, gestational age, urinary creatinine [1]	1.63	0.49	3.33	0.77

[1] Log-transformed.

Table S6. Associations between proline betaine concentration and temporal predictors, only including data above thresholds likely indicative of citrus consumption [1]

**Predictor**	**Beta Coefficient (95% CI)**	***P* value**	**Marginal R-Squared** [2]
Citrus Season (December-May)			
Non-normalized Pro-B	0.14 (-0.16, 0.45)	0.36	0.008
Creatinine-normalized Pro-B	-0.06 (-0.30, 0.18)	0.62	0.003
Gestational Age, days			
Non-normalized Pro-B	-0.0005 (-0.003, -0.002)	0.72	0.001
Creatinine-normalized Pro-B	0.0004 (-0.0015, 0.0022)	0.71	0.001

[1] >100 µM and >30 µmol/mmol creatinine for non-normalized and creatinine-normalized data, respectively.

[2] Proportion of variance explained by the predictor.

Abbreviations: Pro-B, proline betaine

Supplemental Figures

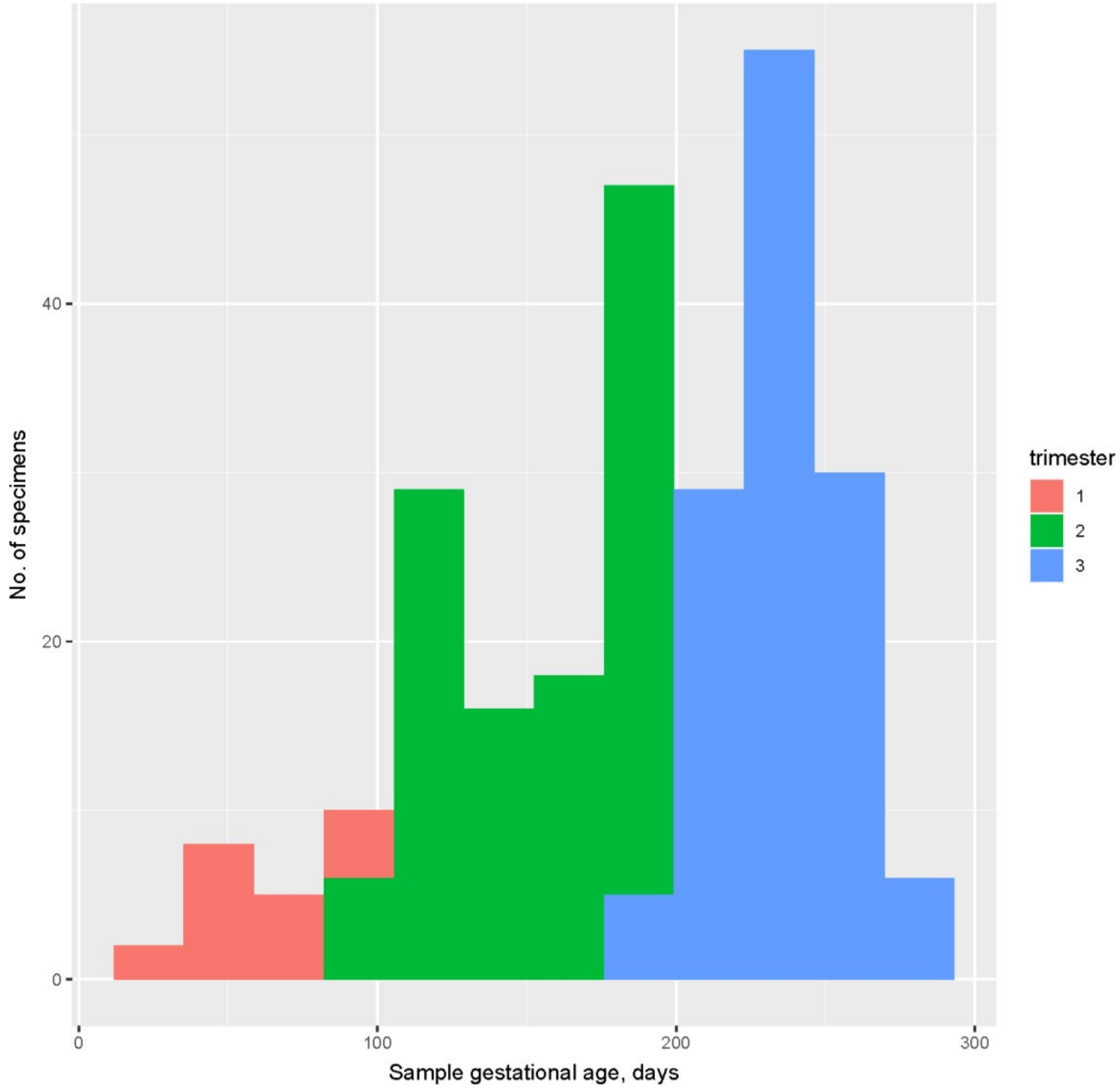

Figure S1. Distribution of gestational age at the time of urine specimen collections

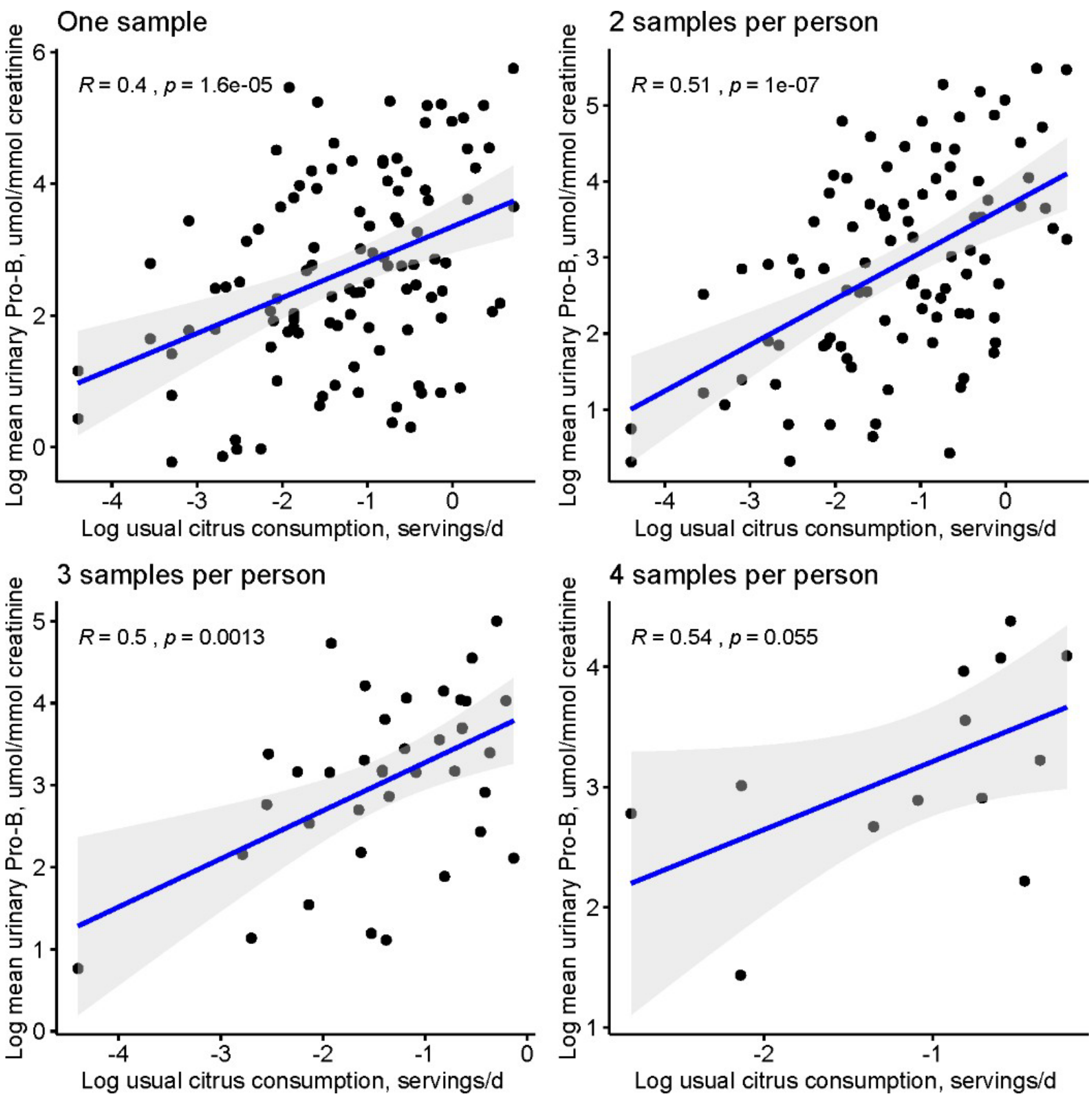

Figure S2. Scatter plots of log urinary proline betaine concentration (μmol/mmol creatinine) by log reported usual citrus intake (servings/day), comparing single sample data with averaged repeated measures. *R* represents Pearson correlation coefficient. Blue lines are derived from linear regression.

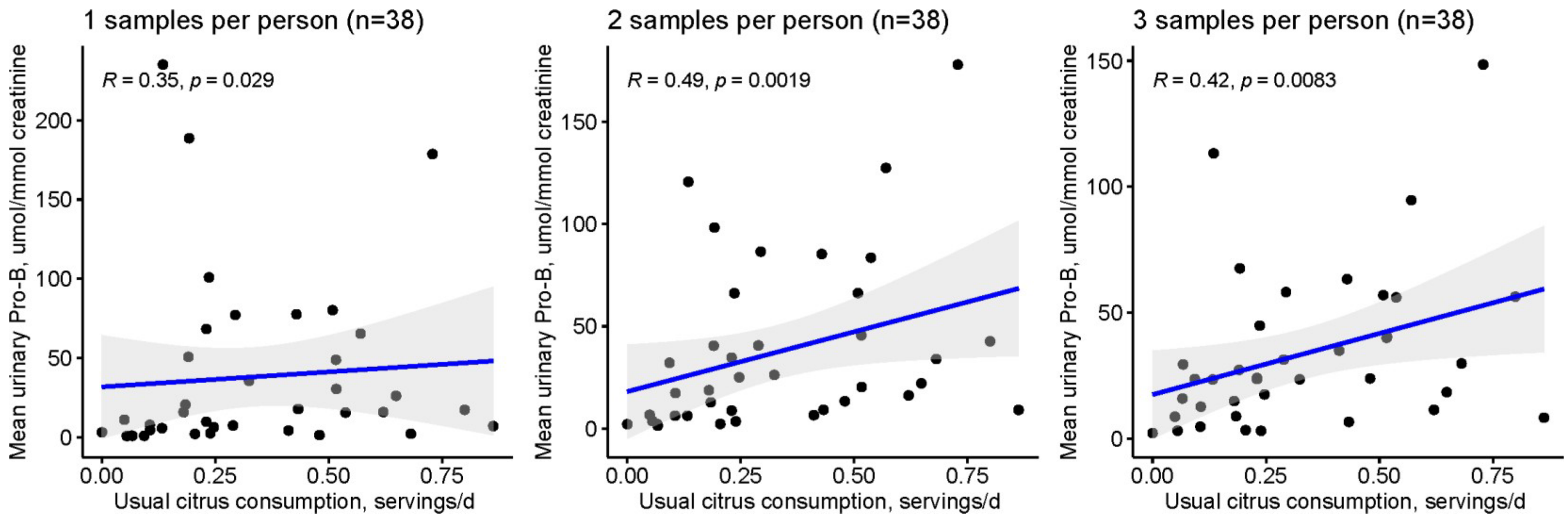

Figure S3. Scatter plots of urinary proline betaine concentration (μmol/mmol creatinine) by reported usual citrus intake (servings/day) including varying numbers of repeated measures, for a subset of individuals with at least 3 samples available. *R* represents Spearman's rank correlation coefficient. Blue lines are derived from linear regression and are included to visualize trends in the data.

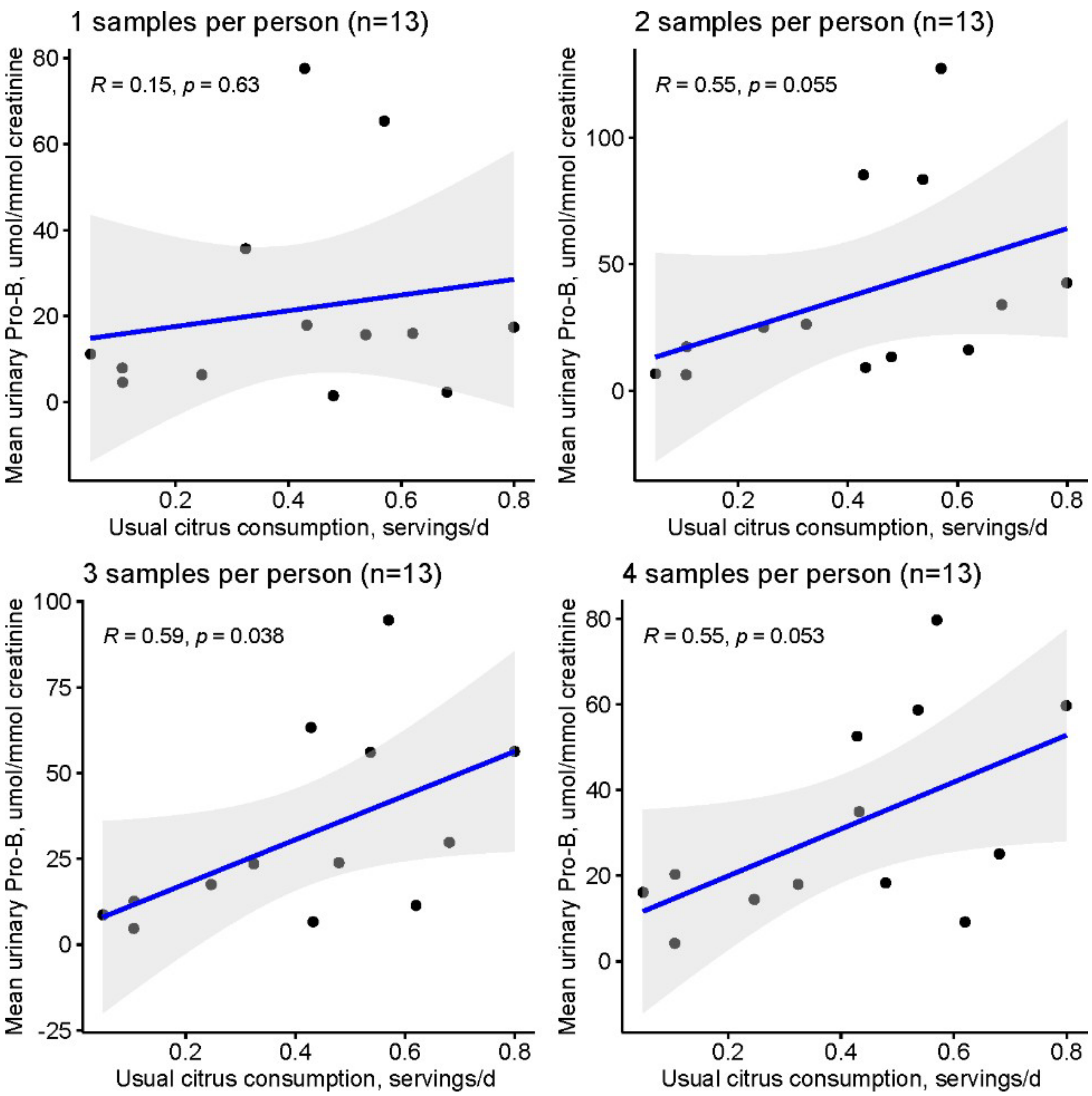

Figure S4. Scatter plots of urinary proline betaine concentration (μmol/mmol creatinine) by reported usual citrus intake (servings/day) including varying numbers of repeated measures, for a subset of individuals with at least 4 samples available. *R* represents Spearman's rank correlation coefficient. Blue lines are derived from linear regression and are included to visualize trends in the data.
